# Perceived Social Risk Scale: development and validation in relation to social status and depression in the UK

**DOI:** 10.1136/bmjopen-2024-092107

**Published:** 2025-10-09

**Authors:** Joseph Newton, Jack L Andrews

**Affiliations:** 1 Cardiff University, Cardiff, UK; 2 Department of Experimental Psychology, University of Oxford, Oxford, UK

**Keywords:** Adolescents, Depression & mood disorders, Psychometrics, Psychosocial Intervention, Psychological Stress

## Abstract

**Abstract:**

**Objective:**

To develop and validate the Perceived Social Risk Scale (PSRS) for assessing perceptions of socially risky behaviours, and to validate it against existing psychological measures such as perceived social status and depressive symptoms in a UK sample of older adolescents and adults.

**Design:**

A cross-sectional study involving exploratory and confirmatory factor analyses.

**Setting:**

Participants were recruited from the Cardiff University’s Department of Psychology participant pool (students completing studies for course credit) and Prolific Academic (a crowdsourcing platform for research volunteers). Data collection occurred between 17 February and 6 May 2024.

**Participants:**

A total of 640 UK participants, including both men and women, aged 18-65.

**Main outcome measures:**

We measured the internal consistency of the PSRS, test-retest reliability and validity against measures including rejection sensitivity, perceived social status, depressive symptoms and resistance to peer influence. Moderation analyses examined the role of perceived social status, age and a sense of belonging in the relationship between PSRS scores and depressive symptoms.

**Results:**

The PSRS showed excellent internal consistency (*α=0.96*) and good test-retest reliability (Intraclass Correlation Coefficient (ICC)*=0.70*). Perceptions of social risks significantly declined with age (*r=−0.20, p<0.001*) and factor analyses confirmed that the PSRS differentiates among four distinct but related social risk constructs: authenticity and integrity (α=0.91), social assertiveness (α=0.72), reservedness (α=0.83) and social non-conformity (α=0.72). For evidence of convergent validity, higher PSRS scores were associated with increased sensitivity to social rejection (*r=0.23, p<0.001),* elevated depressive symptoms (*r=0.13, p=0.012)* and negatively correlated with resistance to peer influence (*r=−0.13, p=0.013*). Local perceived social status significantly moderated the relationship between PSRS scores and depressive symptoms (*β=0.005, SE=0.002, t=2.36, p=0.019*). A general sense of belonging did not moderate this relationship.

**Conclusions:**

Our results confirm that social risk is not a uniform construct but is instead multidimensional. The PSRS offers a reliable and valid tool for assessing multidimensional social risk-taking, with strong internal consistency and test–retest reliability. The interaction between depression and local perceived social status highlights the importance of perceived status on social risk perception.

**STRENGTHS AND LIMITATIONS OF THIS STUDY**

- The Perceived Social Risk Scale (PSRS) was developed and validated using exploratory and confirmatory factor analysis in a large UK sample.
- The PSRS demonstrated strong internal consistency and good test-retest reliability.
- The PSRS was examined against a range of established psychological constructs to assess convergent and divergent validity.
- The PSRS has not been validated in clinical or younger adolescent populations.

## Introduction

 Research on social risk-taking, defined here as any decision or action that could lead to reduced social status or ostracism, such as voicing an unpopular opinion,^1 2^ has received less attention than other risk domains such as health and legal risk-taking. This is surprising given that health and legal risk-taking often occurs in the presence of others and is heavily influenced by social norms.^3^ For example, when deciding whether to take a health or legal risk in the presence of others, individuals are likely to factor in the risk of social rejection or loss of face if they do not engage in a socially desired risk. It has previously been shown that adolescents who perceived higher social benefits from engaging in aggressive and illegal behaviours were significantly more likely to anticipate engaging in those behaviours.^1^ This finding shows that social risk perception is an integral component of *risky* decision-making, as individuals weigh the potential social acceptance or rejection by peers when considering engaging in risky health and legal behaviours.

The majority of work on social risk-taking has focused on adolescence, a period of social reorientation when the peer environment becomes increasingly salient.^2^ Reducing the risk of social exclusion or reduced peer group status during this stage of development is therefore an important goal during adolescence.^1 2^ Indeed, it is well established that adolescents are particularly sensitive to peer influence. Research has found that adolescents exhibit greater susceptibility to peer influence than adults, significantly altering their risk perceptions and behaviours based on peer actions.^2^ Longitudinal studies have demonstrated that susceptibility to peer influence decreases with age as self-regulation and autonomy develop.^4^ Moreover, it has been shown that adolescents’ risk perceptions were significantly influenced by observing peers engage in risky behaviours, with adolescents more likely to downplay risks when it was reported that other peers were taking those risks.^5^ This is supported by other studies which have consistently shown that adolescents are more likely to engage in risky behaviours when peers are present, highlighting the importance of peer dynamics in decision-making.^6 7^

Interestingly, peer influence has also been shown to effectively increase positive behaviours. For example, it has been demonstrated that adolescents displayed more prosocial behaviours when encouraged by their peers,^8^ indicating that peer influence can have a bidirectional effect on both positive and negative behaviours. These behavioural findings can also be understood in the context of findings that adolescents, relative to adults, demonstrate pronounced neural responses to peer rejection, with higher activity in brain regions associated with social pain, such as the anterior cingulate cortex.^9^ In addition, self-reported worry about engaging in social risks declines with age, further highlighting a developmental specificity of these behaviours.^1^

Given that the majority of research on social risk-taking has primarily focused on adolescence, it is not fully understood how social risk perception functions across adulthood. Therefore, in this paper we aimed to develop and validate the Perceived Social Risk Scale (PSRS), a novel tool designed to measure perceived social risk-taking. We validated perceptions of social risk against a number of other measures and then assessed the relationship between depressive symptoms and perceived social status. We aimed to validate this scale for older adolescents and adults (18-65). Through the design of the PSRS for individuals across the lifespan, we are able to gain greater understanding of age-related changes in social risk perception beyond adolescence.

### Depressive symptoms

Social risk-taking is multifaceted and influenced by various factors, including self-perceived social value and depressive symptoms. The Social Risk Hypothesis of Depression^10^ posits that depressive states evolved as an adaptive mechanism to minimise social risk-taking. According to this hypothesis, when individuals perceive themselves as having low social value and high social burden, depressive symptoms function to reduce social engagement, thereby preventing potential social exclusion. Historically, being part of a social group was critical for survival and reproduction,^11^ and therefore depressive symptoms such as social withdrawal and hypersensitivity to social threats serve to mitigate the risk of ostracism and potential exclusion, ultimately aiming to maintain the individual’s social standing within the group.

Empirical evidence provides compelling support for this hypothesis. It is well established that depressive symptoms profoundly affect social behaviour. For example, it has been found that university students with higher depressive symptoms engaged less frequently in social interactions, preferred one-on-one (dyadic) engagements over group settings and often interacted with others who also exhibited depressive symptoms.^12^ Such behaviours could serve to limit negative social evaluations, aligning with the hypothesis that depression-related behaviours aim to mitigate social risk-taking. It has been further demonstrated that individuals who had experienced social exclusion showed a preference for solitude.^13^ This behaviour can be interpreted as a self-protective strategy to reduce exposure to further social threats, consistent with the adaptive function proposed by the social risk hypothesis. In addition, there is evidence that low mood can increase affective theory of mind, suggesting that depressive symptoms may increase orientation towards social information and increase social sensitivity.^14^ This is consistent with the social risk hypothesis, as increased orientation to social cues may improve emotion detection and therefore one’s ability to navigate unstable social situations.^15^

However, recent findings^16^ indicate that individuals with lower self-perceived social value engaged in increased social risk-taking behaviours in a novel social risk-taking task, potentially as a strategy to enhance their social standing or to avoid social exclusion. Interestingly, this proactive approach where individuals take risks to improve their social status or integrate into social groups appears to contrast with the Social Risk Hypothesis of Depression. While the hypothesis posits that individuals with low perceived social value are at greater risk for depression and may adopt risk-averse behaviours to avoid further social devaluation, the observed increas in social risk-taking suggests a different coping strategy. Therefore, while low self-perceived social value can drive both increased social risk-taking to gain social acceptance and socially risk-averse behaviours to avoid further social rejection, these strategies are context-dependent and may be influenced by the presence and severity of depressive symptoms.

It is also important to consider that social withdrawal, a common symptom associated with depressed mood, could be attributed to factors beyond heightened perceived social risk. For instance, reduced energy levels and anhedonia - core symptoms of depression - might inherently limit social engagement^17^ due to diminished motivation and pleasure which impair the ability to sustain relationships. This raises questions about whether social withdrawal reflects an adaptive mechanism or simply the functional outcome of these symptoms. Therefore, further work understanding how the association between social risk perception and depression is influenced by social standing is crucial for disentangling these findings.

### Perceived social status

Self-esteem, perceived social status and hierarchical position are distinct, yet inter-related constructs that influence social behaviour and mental health outcomes. Self-esteem refers to an individual’s subjective evaluation of their own worth, encompassing feelings of competence and personal value.^18^ In contrast, perceived social status reflects one’s self-assessed rank within a social hierarchy, often influenced by comparisons with peers and societal norms.^19^ Hierarchical position, however, denotes an objective placement within a structured system, such as organisational roles or dominance hierarchies, independent of personal evaluations.^20^

Studies have consistently shown that higher self-esteem and perceived social status are linked with lower levels of stress, anxiety and depression, primarily due to stronger social support networks that offer crucial emotional and practical support.^21 22^ For example, individuals with higher social status are more likely to apply for jobs and engage in networking activities.^23^ Higher social status also correlates with healthier behaviours such as regular exercise, balanced nutrition and reduced substance abuse, leading to better overall health outcomes, including improved immune function and lower cardiovascular risk.^24^ These benefits also extend to mental health, with elevated self-esteem and social status being strongly associated with positive mental health outcomes, including lower levels of anxiety and depression and higher levels of life satisfaction and overall well-being.^25^ Furthermore, higher perceived social status often correlates with increased income and improved job selection opportunities. Notably, the Whitehall studies on British civil servants highlighted that individuals in higher occupational grades experienced better health outcomes and longer life expectancies compared with those in lower grades, illustrating the intersection of social status and economic benefits.^26^ Higher status and self-esteem can also enhance social desirability and mating success, as demonstrated in studies which found that individuals perceived as having higher social status were more attractive to potential mates.^27^

Empirical evidence also suggests that higher self-esteem, social status and hierarchical position are correlated with increased risk-taking behaviours. Individuals with elevated self-esteem or social status often perceive themselves as more capable and less vulnerable to the negative consequences of risk, leading to more frequent engagement in risky behaviours. For instance, it was found that individuals who were made to feel powerful were more likely to engage in risk-taking behaviour in economic decision-making tasks.^28^ This propensity has been attributed to an increased sense of control and optimism about outcomes that often accompanies high perceived status. Additionally, it has been shown that men with higher perceived social status were more likely to engage in physically risky behaviours in the presence of attractive women,^29^ suggesting that social status can influence risk-taking in social and mating contexts. Thus, while direct research on the interaction between social risk-taking, depressive symptoms and perceived social status is limited, existing studies on related constructs indicate that higher perceived social status confers numerous advantages across mental, social and physical domains, promoting overall well-being and facilitating a propensity for risk-taking behaviours that can enhance economic and social success.

### Scale rationale

Much of the existing research has focused on adolescence as a period of heightened social risk aversion. However, individuals navigate social risks throughout their lives, constantly weighing the potential benefits against the social costs of their actions. This ongoing negotiation likely involves a sophisticated interplay of perceived social value, mood state, resistance to peer influence, social rejection sensitivity and the desire for social affiliation or approval, which are likely to have varying levels of influence across the lifespan.

In addition, existing work has characterised and measured social risk-taking as a uniform, domain general construct.^1 30^ However, there is reason to hypothesise that social risk taking is multifaceted, comprised of various subdomains. For example, social risks that involve approach behaviours (eg, standing up for an unpopular peer) could be considered conceptually distinct from social risks that involve avoidance behaviours (eg, missing a popular friend’s party), which too could be considered distinct from social risks that violate norms (eg, reading a book during a party).

As such, we can consider social risk as being comprised of multiple domains. *Approach-related* social risks, such as standing up for an unpopular peer or expressing an unpopular opinion, involve confronting group dynamics and prioritising personal authenticity, at the expense of risking social harmony. *Avoidance-related* social risks, such as declining a social invitation or missing a popular event, involve decisions that limit opportunities for social connection and inclusion but serve to protect individual boundaries or preferences. Conversely, social risks which could be considered *norm-violations*, such as stating that you do not like a widely loved celebrity or wearing clothes that your friends would not approve of, involve intentional divergence from group expectations, risking disapproval but asserting individuality. These distinctions highlight a multidimensionality of social risks that prior social risk measurement had not covered.

In the current study, we therefore developed a novel scale to test an individual’s perception of an array of socially risky behaviours. Our scale represents an important evolution from existing measures like the Domain-Specific Risk-Taking Scale (DOSPERT)^30^ and the Health and Social Risk Questionnaire (HSRQ)^1^ for two main reasons. First, we aimed to create a measure that captured a wider variety of socially risky behaviours. For example, the HSRQ^1^ included only seven social risk items and measured social risk as a singular construct. Our scale aims to dissect social risk into specific subcategories, allowing us to make more precise predictions about social risk behaviours. Previous scales have not offered the granularity to draw such distinctions, which is a gap that our scale addresses. Second, by creating a measure that acutely captures social risk perceptions among older adolescents and adults, we will be better able to design studies to assess developmental trajectories of social risk-taking across the lifespan.

As such, to our knowledge, this is the first scale that focuses exclusively on social risk perception. We deliberately designed our scale to capture a broad range of social behaviours to be applicable to a wider demographic. Unlike the DOSPERT, which includes items that may not apply across different age groups or cultural contexts,^1^ the items included in our scale reflect everyday social scenarios that people from diverse backgrounds can understand and evaluate, making the scale more inclusive and applicable for a wide variety of research settings. Therefore, the PSRS addresses this gap by offering a more nuanced instrument to evaluate social risk perceptions among researchers and has the potential to be relevant in clinical contexts.

### Hypotheses

Informed by theoretical work suggesting that social risk is multifaceted, we anticipated that the PSRS would comprise multiple factors. We first ran an exploratory factor analysis (EFA), and consistent with our expectations, the EFA demonstrated that social risk comprised four distinct but related dimensions (see results below). Following this, we preregistered a number of subsequent hypotheses. We first hypothesised that the four-factor structure would be confirmed through confirmatory factor analysis (CFA). We also hypothesised that this four-factor solution would provide a better fit than a one-factor model. Second, we hypothesised that the PSRS would exhibit good concurrent validity by correlating with the social risk subscale of the DOSPERT^30^ and the Online and Offline Social Sensitivity Scale (O^2^S^3^).^31^ Third, we hypothesised the PSRS to demonstrate good convergent validity with depressive symptoms (measured by the Depression Anxiety Stress Scales (DASS)-7),^32^ the Resistance to Peer Influence Scale,^33^ perceived social status^21^ and belonging.^34^ Finally, we hypothesised that PSRS scores would negatively correlate with age, such that older individuals would report lower social risks perception than younger individuals.

Additionally, we hypothesised that depressive symptoms would moderate the relationship between perceived social risk and age, with those experiencing higher depressive symptoms perceiving greater social risk across all ages. We also hypothesised that perceived social status would moderate the relationship between perceived social risk and depression, with a stronger relationship for individuals lower in objective income. Lastly, we hypothesised that belonging would moderate the relationship between perceived social risk and depression, with weaker associations among those reporting greater feelings of belonging.

### Consent

All participants in both samples provided informed written consent electronically before taking part in the experiment. Participants were provided with detailed information on the purpose of the study, procedures and their rights as participants, in line with Cardiff University’s ethical guidelines, prior to providing consent.

### Open science statement

This design, hypothesis and analysis plan were preregistered prior to data collection: https://osf.io/97ure/?view_only=b7090d9e244540babd028648d753f3de. In this paper, we report all measures, manipulations and data exclusions. The R script and data are available from the corresponding author on reasonable request.

## Method

### Patient and public involvement

No patient or public involvement was used in this research.

### Participants: sampling and recruitment

A convenience sampling approach was used, with participants (N=640) recruited from two sources: (1) the Cardiff University Psychology participant pool, and (2) Prolific Academic, an online platform that offers a recruitment of a UK-based adult sample. This approach allowed for the inclusion of both younger adults (18–24) from the university setting and a wider, more diverse age range (18–65 years) from the general UK population.

### Inclusion and exclusion criteria

We did not specify any specific inclusion or exclusion criteria beyond the requirement that participants be aged 18–65, UK based and capable of completing an online survey. We did, however, include four attention checks that participants had to pass to be included in the sample.

### Sample size

Sample size for our EFA was determined based on Kaiser-Meyer-Olkin (KMO) index and the Bartlett’s test, with scores above 0.7 and below 0.05, indicating a suitable sample size, respectively. Our CFA sample size was calculated a priori and preregistered. We aimed for a minimum of 360 participants (10 participants per question included in the EFA). In practice, we over-recruited to account for attrition and poor responding.^35^,^37^

#### Sample 1: exploratory factor analysis

Participants (n=251) were recruited from the Cardiff University Psychology participant pool. Participants (211 women, 38 men, 1 non-binary) were aged 18–25 years (mean age=19.51, SD=1.18). Participants took part in return for course credit. No participants were excluded from the data analysis.

#### Sample 2: confirmatory factor analysis and validation

Participants (N=389) were recruited from Prolific Academic, all residing in the UK. Participants (243 women, 143 men, 3 non-binary) were aged 18-65 years (mean age=39.33, SD=11.68). The majority identified as white (80.2%), followed by Asian, including Pacific Islander (8.1%), biracial or multiracial (5.3%), black or African American (5.1%) and Hispanic (1.3%). Regarding socioeconomic status, 13.5% of participants reported an annual household income of less than £11 999, 25.4% reported £12 000-24 999, 44.7% fell within £25 000-49 999, 11.4% reported incomes of £50 000-74 999 and 2.3% had an income of £75 000 or more. Additionally, 2.7% of participants preferred not to disclose their income. Participants were paid in line with Prolific’s fair and ethical payment scheme. Two participants were excluded for failing attention checks.

### Questionnaire development: Perceived Social Risk Scale

To assess the degree to which individuals perceive the risk of engaging in social risk behaviours, we developed the PSRS. Initially, we drew certain items from existing measures of social risk, namely the HSRQ^1^ and the DOSPERT.^30^ We reviewed each social risk item in both scales and hypothesised that there would be different underlying dimensions between some questions on both scales (eg, approach vs avoidance behaviours). We then supplemented these items by comprehensively reviewing the theoretical and empirical literature on social risk and related constructs, including examining research and measures related to belongingness,^34 38^ rejection sensitivity^31 39^ and resistance to peer influence,^33^ ensuring a broad conceptual foundation for item generation. Finally, items were carefully crafted using clear and accessible language, avoiding jargon or culturally specific references. This approach prioritised scenarios that were broadly relatable, such as ‘Not laughing at a group’s inside joke that you don’t find funny’, rather than situations tied to specific cultural norms.

Items were constructed to reflect a wide array of social risk behaviours. For instance, social risk items included statements such as: ‘Telling a risky joke’ (approach-related social risk), ‘Wearing clothes that your friends wouldn’t approve of’ (norm-violating social risk), ‘Not contributing to a group gift that you find too expensive’ (avoidance-related social risk) and ‘Refusing to spread a rumour, even when all your friends are doing it’ (norm-violating social risk). As such, a list of 36 items was developed and created to create a list of social risk scenarios that could tap into distinct constructs of social risk-taking. The list was designed to be suitable across a series of social risk situations, aiming to ensure that each item was distinct and appropriately targeted. In the questionnaire administered to participants, individuals were instructed: ‘For each statement, please rate how much risk you would feel engaging in this behaviour’. Responses were recorded on a sliding scale from ‘Not at all risky (1)’ to ‘Very risky (7)’. The questionnaire was administered online, with numerical values (1–7) visible along the slider (see online supplemental table 1 for the original questionnaire).

### Measures

*Depressive symptoms*: The seven-item depression subscale of the short version of the DASS-21^32^ was administered to assess symptoms of depression. The DASS-7 was selected for its reliability in measuring depressive symptoms and its ability to differentiate depressed mood from anxiety and stress. The conciseness and empirical validity of the DASS-7 ensured a reduction in participant burden, making it well-suited for online studies. Additionally, its use in prior research on rejection sensitivity and depression^31^ ensures methodological consistency and comparability, in which we also administered the Rejection Sensitivity Questionnaire (O^2^S^3^) from the same source to maintain comparability of measures. This scale asks seven questions about how much various scenarios applied to individuals over the last 7 days. The scale ranges from 0 (did not apply to me at all) to 3 (applied to me very much, or most of the time). α=0.93.

*Perceived social status*: The McArthur Scale of Subjective Social Status^21^ asks participants to place themselves on a ‘social ladder’, representing their social status relative to others. The scale ranges from 1 (lowest perceived social status) to 10 (highest perceived social status). Participants rate their perceived social status in two contexts: locally, within their own communal network, and nationally, in comparison to the broader society. α=0.85.

*Resistance to peer influence*: The Resistance to Peer Influence scale (RPI)^33^ is a 10-item scale measuring an individual’s resistance to peer influence. Participants choose the option that best describes their group (more or less peer-resistant) and indicate the degree to which they feel they belong to this group (‘Really true’ vs ‘Sort of true’). α=0.76.

*Belongingness*: The General Belongingness Scale (GBS)^34^ is a 12-item questionnaire assessing feelings of belonging and acceptance within social groups. The scale has a two-factor structure that measures acceptance and inclusion, as well as rejection and exclusion. Items are rated on a 7-point scale from 1 (strongly agree) to 5 (strongly disagree). Belonging α=0.95, rejection subscale α=0.93, overall α=0.55.

*Social risk-taking*: The six-item social risk scale of the DOSPERT^30^ measures existing correlations against already existing measures of social risk. This scale assesses the likelihood of engaging in social risk-taking on a 7-point Likert scale, where 1 indicates no willingness to take risks and 7 indicates high willingness. α=0.65.

*Rejection sensitivity*: The O^2^S^3^^31^ is an 18-item scale that is an update on previous rejection sensitivity measures. The O^2^S^3^ allows for a measurement of rejection sensitivity in both an online and offline context. Participants respond on a 4-point Likert scale (strongly disagree-strongly agree). α=0.87.

#### Procedure

The EFA Sample 1 completed the 36-item PSRS through the Cardiff University online Psychology system. Following informed consent, the questionnaire was administered online. After completion of the survey, participants were awarded course credit for their completion. Following this, we then ran CFA Sample 2 through Prolific Academic.

##### Statistical analysis

All data were analysed using R (V.2024.04.1),^40^ with key packages such as *lavaan, lmTest* and *psych*.

##### Exploratory and confirmatory factor analysis

We first conducted an EFA using oblique (oblimin) rotation on the initial 36 items relating to health and social risks on a sample of 251 Cardiff University students. We determined the suitability of our sample size and data for EFA based on the KMO index (>0.70) and Bartlett’s test (<0.05).^41^ We determined the number of factors to retain based on examination of the scree plot, retention of factors with eigenvalues of 1 or greater and factors with at least three items. Items with factor loadings of <0.4 were removed. Following factor and item reduction based on the above criteria, we subjected the same data to a CFA to assess the fit of the proposed factor structure.

We then used CFA to assess the strength of this factor structure in a new adult sample of 389 adults from Prolific Academic. Our primary, preregistered, measure of model fit was root mean squared error of approximation (RMSEA). An RMSEA of around <0.08 indicates reasonable fit.^42^ We also assessed the model fit with the standardised root mean square residual (SRMR; <0.08 reasonable fit), Comparative Fit Index (CFI; >0.9 reasonable fit) and the Tucker-Lewis Index (TLI; >0.9 reasonable fit). We computed measures of internal consistency using Cronbach’s alpha and McDonald’s omega. We further tested the fit of each four-factor CFA using Akaike Information Criterion (AIC), by comparing a one factor solution (where all items are loaded onto one higher order risk factor) with the four-factor solution, in which a lower AIC represents a better fit to the data. We then assessed test–retest reliability of the PSRS by inviting 108 participants from the adult CFA sample to complete the PSRS again 13–15 days after the first completion.

##### Validation and test–retest reliability

To assess concurrent validity, we assessed the relationship between the PSRS, online rejection sensitivity,^31^ social risk-taking^30^ and resistance to peer influence.^33^ To assess convergent validity, we assessed the PSRS against existing measures of depressive symptoms,^32^ using Pearson r correlations. These analyses were intended to provide evidence of convergent and divergent validity, rather than agreement. To establish the test–retest reliability of the PSRS, we invited 110 participants from the CFA sample to complete the PSRS questionnaire a second time 13–15 days after the first completion. We then used the Intraclass Correlation Coefficient (ICC) to establish the relationship between these individuals’ scores at time point 1 and 2.

### Sample 1: exploratory factor analysis

Analysis showed that the sample size (n=251) was suitable for conducting factor analysis (KMO=0.94, Bartlett’s test<0.001). Three factors showed eigenvalues above our threshold of one: 13.927, 1.915 and 1.485. Despite the fourth factor having an eigenvalue of 0.900, we decided to retain it for theoretical reasons and due to the scree plot suggesting a four-factor solution. This decision was also guided by the theoretical importance of the construct it represents, which we argue is an essential aspect of social risk. This fourth factor captures the dimension of social assertiveness, which taps into a crucial component of social risk, helping us measure behaviours that involve confidently expressing one’s opinions or rights, even when it deviates from social norms. In sum, this resulted in a four-factor, 27-item solution. The Cronbach’s alpha was α=0.96, indicating excellent internal consistency. A description of the four factors is provided below in table 1.

**Table 1:** Retained factors, description and example items

Name of factor	Description	Example item
Authenticity and integrity	Emphasising honesty, personal values and staying true to oneself	‘Refusing to spread a rumour, even when all your friends are doing it’.
Social assertiveness	Confidently standing up for beliefs in social situations	‘Not contributing to a group gift that you find too expensive’.
Reservedness	A preference for solitary activities, as opposed to social ones	‘Choosing to stay in on the weekend instead of going out with friends’.
Social non-conformity	Engaging in behaviours that may not align with social norms	‘Defending an unpopular opinion that your friends don’t believe in’.

### Item reduction

Additional steps were taken to refine the questionnaire. First, items that did not load strongly onto any of the four factors were removed, resulting in the elimination of nine items that loaded below 0.40 on any factor. Further, items that cross-loaded onto two or more factors were judged on each individual basis as to which factor they would be most conceptually attributed to. To create a succinct and meaningful scale, items representing each factor were retained based on their loading strength and theoretical relevance, leading to a final 27-item scale. This four-factor solution represented a better fit (CFI=0.884, TLI=0.872, SRMR=0.061 and RMSEA=0.073, AIC=19 024.363) compared with the three-factor model, which yielded CFI=0.831, TLI=0.819, SRMR=0.071 and RMSEA=0.081, AIC=22 733.795.

### Sample 2: confirmatory factor analysis (Sample 2—Adults)

We then conducted a CFA on a new sample of 389 adults. The sample size was based on general methodological rulings suggesting that 10 participants per item is appropriate.^43^ With 27 items, we aimed for 270 participants and over-recruited to increase the robustness of our results and to account for incorrect responses or missing data. The four-factor structure adequately fit the data according to our primary fit index; RMSEA=0.08 (0.07–0.09). Other fit indices were good (SRMR=0.06) or fell just below the suggested cut-off (CFI=0.87) and (TLI=0.83). For the CFA, Cronbach’s alpha was α=0.92 and McDonald’s omega was ω=0.94, further confirming the reliability of the scale across different samples and analyses. Additional analysis confirmed the reliability of the subscales: Factor 1**=***Authenticity and integrity,* α=0.91. Factor 2=*Social assertiveness,* α=0.72. Factor 3=*Reservedness.* α=0.83. Factor 4 (PSRS)=*Social non-conformity,* α=0.72. Higher scores indicate a greater social risk perception. The final 27 items and their corresponding factors are shown below in table 2.

**Table 2:** The Perceived Social Risk Scale (PSRS)

Item	Statement	Factor
1	Voicing an unpopular opinion	4
2	Telling a risky joke	4
3	Wearing clothes that your friends would not approve of	3
4	Missing a popular friends’ party	3
5	Defending an unpopular opinion that your friends do not believe in	4
6	Not drinking alcohol at a social event where everyone else is	3
7	Spending the weekend alone despite friends wanting to hang out	3
8	Not laughing at a group’s inside joke that you do not find funny	3
9	Skipping a popular movie night to watch a documentary instead	3
10	Admitting you have not seen a popular TV show that everyone is talking about	1
11	Reading a book at a social event instead of mingling	3
12	Quitting a popular activity because it is no longer enjoyable for you	1
13	Not contributing to a group gift that you find too expensive	2
14	Refusing to spread a rumour, even when all your friends are doing it	1
15	Choosing to stay in on the weekend instead of going out with friends	3
16	Refusing to participate in a prank that you feel is in poor taste	1
17	Bringing up a serious topic of discussion at a light-hearted gathering	2
18	Not gossiping about someone when your friends are too	1
19	Standing up for a political belief that is unpopular within your friend group	2
20	Declining to cheat on a test, even when friends assure you it is safe	1
21	Choosing to walk away from a group that is mocking someone else	1
22	Not altering your appearance when it is the norm in your group	1
23	Stating that you do not like a widely loved celebrity	1
24	Refusing to change your opinion just to fit in with a group discussion	1
25	Not using words or phrases that everyone else is using because you do not like it	1
26	Choosing a different meal at a restaurant when all of your friends are having the same	1
27	Not lying to authority figures when your peers are encouraging you to	1

For each statement of the PSRS, individuals indicate how much risk that they perceive on a 7-point Likert scale that goes from 1—Not all risky, to 7—very risky. Factor 1 (PSRS)=*Authenticity and integrity*. Factor 2 (PSRS)=*Social assertiveness***. **Factor 3 (PSRS)=*Reservedness*. Factor 4 (PSRS)=*Social non-conformity***. **Higher scores indicate a greater social risk perception.

### Test–retest reliability

To measure the test-retest reliability of the PSRS, 108 adult participants were invited to complete the questionnaire a second time 13-15 days later; 108 participants responded. Mean age=40.72, SD age=11.17; 42 were male, 66 were female. Test-retest reliability for the overall scale across the interval was good, *ICC=0.70 (95% CI 0.571 to 0.797*). This confirms the stability of the scale items over time. The subscale specific test-retest values are included in the online supplemental material 4.

### Cross-sample consistency

We then compared responses across participants from the EFA and CFA based on age. Specifically, to ensure consistency between our University sample and our Prolific sample, we tested how participants with the same age (18-25) responded. The results indicated excellent consistency between University and Prolific sample, *ICC=0.95 (95% CI 0.944 to 0.957*).

### Validation

To assess convergent and concurrent validity, participants also completed measures of online and offline rejection sensitivity (O^2^S^3^), depressive symptoms (DASS), belongingness (GBS), resistance to peer influence (RPI) and subjective social status (MacArthur Scale). We correlated overall PSRS score with these measures and also reported correlations among the subscales of the PSRS with these measures. Data visualisations of each association are included in the (online supplemental material, figures S1–S8). Correlations between the PSRS individual subscales and other measures are reported below and in full in the (online supplemental table S2).

#### Association with rejection sensitivity

The PSRS positively correlated with online and offline rejection sensitivity as measured by the O^2^S^3^ (*r(387)=0.23, p<0.001, 95% CI 0.13 to 0.32),* such that individuals who scored high on perceived social risk also scored high on rejection sensitivity.

#### Association with depressive symptoms

The PSRS positively correlated with depressive symptoms as measured by the DASS (*r(349)=0.13, p=0.012, 95% CI 0.03 to 0.23*). Confirming that individuals who scored highly on perceptions of social risk also reported higher levels of depressive symptoms.

#### Association with social risk-taking

The PSRS negatively correlated with the likelihood of engaging in social risks as measured by the social risk scale of the DOSPERT (*r(387)=−0.15, p=0.003, 95% CI −0.24 to –0.06). Therefore,* individuals who scored highly on perceived social risk were less likely to engage in social risk behaviours.

#### Association with belonging

The PSRS negatively correlated with the GBS *(r(386)=−0.15, p=0.002, 95% CI −0.24 to –0.06),* indicating that individuals who perceive higher social risks feel less of a sense of belonging within their social groups.

#### Association with subjective social status

The PSRS was examined in relation to subjective social status using the MacArthur Scales for both local and national contexts (ie, how people perceive their social status relative to those around them, and relative to everyone in their country). The correlation between the PSRS and MacArthur Local scale was negative but not statistically significant (*r(386)=−0.08, p=0.116, 95% CI −0.17 to 0.01) as was the* correlation with the National scale (*r(386)=−0.06, p=0.268, 95% CI −0.15 to 0.03*).

#### Association with resistance to peer influence

The PSRS negatively correlated with the RPI (*r(387)=−0.13, p=0.013, 95% CI −0.22 to –0.03*). This suggests that individuals with a higher social risk perception reported greater susceptibility to peer influence.

#### Association with age

The PSRS demonstrated a significant negative correlation with age. Age negatively correlated with PSRS scores (*r(387)=−0.20, p<0.001, 95% CI −0.29 to –0.11),* indicating that older individuals perceive less social risk than younger individuals.

## Moderation analysis

### H1: depressive symptoms will moderate the relationship between PSRS and age

To investigate whether depressive symptoms moderate the relationship between perceived social risk (PSRS) and age, we conducted a regression analysis that included an interaction term between age and depressive symptoms (DASS score). The regression model found that the interaction term between age and depressive symptoms was not statistically significant (*β=0.000, SE=0.000, t=1.093, p=0.275*). This suggests that depressive symptoms did not significantly moderate the relationship between PSRS and age. However, we found a main effect of age (*β=−0.015, SE=0.005, t=12.949, p=0.005*), such that perceived social risk decreases with age. These results suggest that as individuals become older, their perception of social risk decreases, irrespective of their levels of depressive symptoms. The full regression output for H1, H2 and H3 is included in the online supplemental material, tables S1–S6.

### H2: perceived social status will moderate the relationship between PSRS and depressive symptoms, while controlling for income

To examine whether perceived social status (at the national and local level) moderates the relationship between perceived social risk (PSRS) scores and depressive symptoms (DASS score), we conducted a regression analysis that included an interaction term between perceived social status and depressive symptoms. We also controlled for actual income.

**Figure 1:**
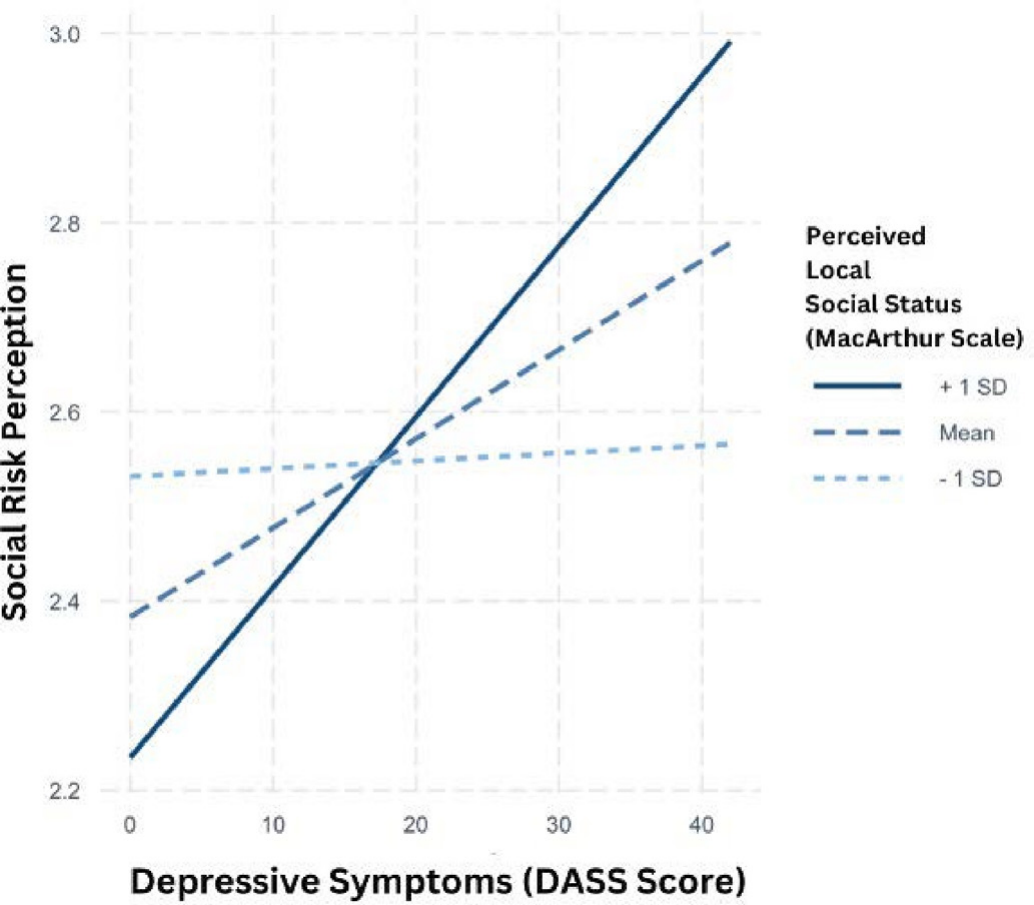
Interaction plot illustrating the relationship between depressive symptoms (measured by DASS Score) and social risk perception (PSRS scores), moderated by perceived local social status (MacArthur Scale). The plot shows social risk perception at three levels of perceived local social status: +1 SD (solid line), mean (dashed line) and −1 SD (dotted line). DASS, Depression Anxiety Stress Scales; PSRS, Perceived Social Risk Scale.

Perceived social status at the national level did not moderate the relationship between PSRS and depression. Specifically, we found a non-significant interaction between national perceived social status and depressive symptoms (*β=0.004, SE=0.002, t=1.791, p=0.074*). However, perceived social status at the local level did moderate this relationship. We found a significant interaction between local perceived social status and depressive symptoms, while controlling for income (*β=0.005, SE=0.002, t=2.360, p=0.019*). These findings suggest that local perceived social status significantly moderates the relationship between PSRS and depressive symptoms. That is, individuals with higher local perceived social status show a stronger relationship between depressive symptoms and perceived social risk. Additional Simple Slope Analyses showed that at one SD below the mean of local perceived social status (−1 SD, 3.77), the slope was not significant (*β=0.00, SE=0.00, t=0.17, p=0.86*). At the mean level of local perceived social status (5.40), the slope was significant (*β=0.01, SE=0.00, t=2.36, p=0.02*). At one SD above the mean (+1 SD, 7.03), the slope was significant (*β=0.02, SE=0.01, t=3.10, p<0.001*). This highlights that the relationship between depression and perceived social risk is stronger among individuals with higher perceived local social status, relative to those who perceived their local social status as lower. This interaction is visualised in figure 1.

### H3: a sense of belonging will moderate the relationship between PSRS and depressive symptoms

To examine whether a sense of belonging moderates the relationship between perceived social risk (PSRS) scores and depressive symptoms (DASS total score), we conducted a regression analysis that included an interaction term between total sense of belonging and depressive symptoms. We found a non-significant interaction between a sense of belonging and depressive symptoms *(β=0.001, SE=0.002, t=0.270, p=0.787*). This suggests that the sense of belonging did not significantly moderate the relationship between PSRS and depressive symptoms. All main effects were also non-significant.

## Discussion

In this study, we developed and validated the PSRS to enhance and measure our understanding of social risk. Existing research has typically conceptualised social risk as a uniform construct. However, we hypothesised and demonstrated that perceptions of social risk-taking are dimensional and can be categorised into a number of related but distinct categories.

The exploratory and confirmatory factor analyses revealed a robust four-factor structure for the PSRS, encompassing the following domains of social risk: (1) *authenticity and integrity*, (2) *social assertiveness*, (3) *reservedness* and (4) *social non-conformity*. These findings reflect the complexity of social interactions and the varied contexts in which individuals perceive social risks. The PSRS demonstrated excellent internal consistency and good test-retest reliability, indicating its acceptability as a trait measure of social risk perception. While our results supported a multidimensional account of social risk which broadly aligned with our predictions, our findings indicated that certain factors were more nuanced than we anticipated. For example, rather than a single factor capturing norm-violating behaviours, our results suggested a meaningful distinction between social assertiveness, which involves confidently advocating for personal beliefs in social settings, and non-conformity, which captures risks associated with behaviours that diverge from group norms. This suggests that while both assertiveness and non-conformity involve some degree of standing out from the group, they may be distinct in how individuals perceive the social consequences of these actions.

The PSRS demonstrated strong convergent validity, evidenced by significant correlations with established measures such as the O^2^S^3^. Higher PSRS scores were associated with increased social rejection sensitivity, which aligns with existing research indicating that individuals perceiving higher levels of social risk are more sensitive to rejection.^1 2 44^ This relationship can be understood through the lens of social anxiety and interpersonal sensitivity theories, which suggest that individuals who perceive themselves as vulnerable to social exclusion are hyperaware of social cues indicating potential rejection.^39 45^ Individuals, therefore, constantly monitor their social environment for signs of exclusion or disapproval, thereby exacerbating their perceived social risks.

Additionally, the PSRS showed a positive correlation with depressive symptoms, aligning with the Social Risk Hypothesis of Depression. This hypothesis suggests that heightened sensitivity to social risks is associated with an increased degree of depressive symptoms.^10^ The underlying mechanism here involves the evolutionary perspective that depressive states may serve as an adaptive response to perceived social threats. When individuals feel that their social value is low, they may exhibit depressive symptoms such as social withdrawal and heightened sensitivity to social threats to avoid further social devaluation and potential exclusion.^46 47^ Historically, this adaptive function may have served individuals to avoid behaviours that might lead to social rejection, thereby preserving their social bonds and overall survival within a group context. However, it is important to note that this process is functional at the lower end of the depressive spectrum. That is, when low mood or social withdrawal is short term and transient. Chronic, long-term, low mood and associated depressive symptoms are interpreted as a maladaptation of this system.^10^

Moreover, consistent with developmental theories^2 48 49^ and previous work,^1^ our findings show that perceived social risk decreases with age. Younger individuals, particularly adolescents, are more sensitive to social risks, which can be attributed to the heightened importance of peer relationships and social status during this developmental period.^50^ This trend underscores the importance of considering age when assessing social risk perceptions and when considering periods of development that may benefit most from interventions related to perceiving and engaging in social risks.

Interestingly, we found that local perceived social status (ie, within one’s immediate community) significantly moderated the relationship between PSRS scores and depressive symptoms, while national perceived social status (ie, within the broader society) did not. This suggests that immediate social environments play a more crucial role in influencing how depressive symptoms relate to perceived social risk. The significance of local social status may be due to direct, day-to-day interactions and immediate feedback from close social circles, impacting mental health and life satisfaction.^22 24^ National social status, potentially being more abstract, may have less impact on immediate social experiences, explaining the lack of a significant moderating effect in our study. These findings align with research showing that local social determinants, especially immediate relationships, impact mental health and well-being.^51^ Immediate social relationships, such as those with family, friends and close community members, can provide essential emotional support, practical assistance and a sense of belonging,^22 24^ as well as buffering against stress and promoting psychological resilience.^52^ Other findings have confirmed that more immediate social relationships have a greater effect on mental health outcomes, relative to broader national perceptions of social status and social belonging, which tend to be more abstract and less connected to an individual’s daily experiences.^21^ Thus, the quality and stability of close personal relationships shape perceptions of social risk and their psychological impacts.

Counter to our hypothesis, we found that a general sense of belonging did not significantly moderate the relationship between PSRS and depressive symptoms. One possible explanation for our finding that a general sense of belonging did not impact the relationship between social risk perception and depression is that an individual may feel generally included in their social environment yet still perceive particular social actions as risky. This suggests that belonging alone may be insufficient to mitigate the distress associated with high social risk perception, underscoring the need to consider other factors when examining how social risk and depression intersect.

### Subscale correlations

In line with our hypothesis, analyses of the PSRS confirm that social risk is not a uniform dimension but comprises distinct and meaningful categories. The authenticity and integrity subscale showed no significant association with depression or perceived social status, suggesting that high levels of personal integrity may not have a direct impact on depressive symptoms or social valuation. The social assertiveness subscale was significantly positively associated with depressive symptoms, indicative of the potential psychological costs associated with social confrontation. However, this subscale did not show a significant association with perceived social status, suggesting that assertive behaviours might not influence how individuals perceive their social standing. Similarly, the social non-conformity subscale was significantly positively associated with depressive symptoms, indicating a direct link between non-conformist behaviours and increased psychological distress. However, similar to the authenticity and integrity subscale, the reservedness subscale showed no significant association with depression or perceived social status, highlighting that reserved behaviour did not affect psychological distress or social valuation. The reservedness subscale also showed a significant negative association with perceived social status, suggesting that individuals engaging in non-conformity might feel themselves to be less favourably perceived within their social groups. Our findings underscore the distinctiveness of each subscale, illustrating how various dimensions of social risk relate differently to psychological constructs like depression and perceived social status. Therefore, the PSRS effectively distinguishes between different types of social risk perception and their associations with depression and other related constructs.

### Implications for future research and interventions

The PSRS provides a valuable tool for researchers to assess an individual’s perception of social risk-taking. By distilling social risk into discrete subcategories, our scale facilitates the measurement of the specific social risks that individuals perceive and the factors that influence these perceptions. Future research should explore these distinct elements of social risk in more diverse and representative samples to enhance the generalisability of the findings. If future research establishes robust psychometric properties of the PSRS in clinical contexts, clinicians may eventually consider using it to better understand social risk perceptions that contribute to maladaptive behaviours or distress. For example, clinicians could use the PSRS to identify individuals with heightened sensitivity to social risks, a feature often linked to conditions such as social anxiety, depression and interpersonal difficulties. This information could guide therapeutic approaches by highlighting specific risk perceptions that contribute to maladaptive behaviours or distress. However, until then, the PSRS should be regarded primarily as a research measure rather than a clinical assessment instrument.

Additionally, while the PSRS did not show significant direct correlations with perceived social status, the interaction between local perceived social status and depression offers potential for targeted interventions. Enhancing local social support and addressing negative social experiences could mitigate the impact of perceived social risks on mental health, particularly for adolescents and other vulnerable populations. Reducing social risk perceptions in certain contexts is crucial as high perceived social risks are a likely contributor to heightened anxiety, avoidance behaviours and social withdrawal, which can be detrimental to mental health.^53^ By reducing these perceptions, individuals are more likely to engage positively in social interactions and foster a sense of belonging and well-being. This is particularly important during sensitive periods of development, such as adolescence, when individuals are highly sensitive to peer influence and social dynamics.

Most existing research on developmental shifts in social risk-taking has concentrated on adolescence to early adulthood (ages 10-24), leaving little known about how social risk perception evolves in later adult years. Consequently, our finding that perceived social risk declines across the 18-65 age range offers a novel insight into an underexplored period of development. These results warrant further investigation into how factors such as shifting life roles, accumulating social experiences (such as rejection) or changing social priorities influence social risk perception into mid-adulthood and late-adulthood.

### Limitations

It is important to note several limitations of the PSRS. While the PSRS aims to capture a broad range of social risk behaviours, it may not fully capture all types of social risk. Additionally, this study is the first to empirically show that social risk is comprised of distinct categories. Although the factor structure identified through exploratory and confirmatory factor analyses appears robust, it is crucial for future research to replicate these findings in different and more diverse samples. Replication studies will help confirm the generalisability and stability of the factor structure across various populations. In addition, the PSRS was not validated in a younger adolescent population. Given that adolescence is a sensitive period for social development and risk-taking behaviours, future studies should focus on validating the PSRS in adolescent samples. This would help determine the scale’s applicability and relevance for younger individuals.

One limitation of our measure is that, although we endeavoured to construct items that were broadly relevant and accessible by avoiding jargon or culturally specific references, these steps were not systematically tested for applicability across different cultures or demographic groups. While the scenarios were designed to reflect everyday social situations, it is possible that certain items may still be influenced by cultural norms, language nuances or social contexts that were not fully captured in our sample. Consequently, future research should undertake cross-cultural validation and further diversify sampling to confirm the scale’s relevance and comprehensiveness in more varied populations.

In addition, while the correlations between the PSRS and related constructs were statistically significant, the strength of these associations ranged from small to moderate. Importantly, as there are no direct comparator scales, our correlations with related constructs should be interpreted as validity evidence rather than agreement. These effect sizes are consistent with theoretical expectations, as social risk perception is likely influenced by a multitude of factors beyond the constructs examined in this study, such as further individual differences (eg, neuroticism), the cultural context (eg, collectivistic vs individualistic norms), prior experiences of social acceptance or rejection, situational aspects of the peer group and any specific mental health conditions (such as mood disorders). While these findings provide preliminary evidence of the PSRS’s validity, they highlight the need for further research to explore additional predictors and outcomes of social risk perception.

It is also important to consider that while the factors derived from our analysis provide insight into the dimensions of social risk, we acknowledge that they may appear less intuitive due to overlaps in context (eg, solo vs social activities) or action type (eg, action vs inaction). Future research could further refine these factors by exploring context-specific distinctions or alternative theoretical frameworks, such as the role of action versus inaction in shaping responses to social risk-taking.

Another point of difference between the PSRS and the DOSPERT^30^ is that the focus of the PSRS is on risk perception rather than a self-reported willingness to engage in social risk-taking behaviours. While perceived risk and willingness to engage in risk-taking behaviours are conceptually related, they are distinct constructs, and it remains unclear whether differences in perceived social risk translate into differences in behavioural risk-taking. The absence of a direct measure of willingness to engage in social risks in this study limits our ability to draw conclusions about behavioural outcomes, particularly given the proposed links between depressive symptoms and actual risk-taking behaviours.

The EFA was conducted in a university sample (1825) to identify the underlying factor structure of the PSRS in a demographic for whom social risk is particularly salient,^2^ which makes them an appropriate population for uncovering the foundational structure of the scale. The CFA, in contrast, was conducted in a broader age range (18–65 years) to test the generalisability of the identified factor structure across a wider population. While this approach provides valuable insights into the robustness of the PSRS, it introduces the possibility that age-related differences in social risk perception could influence the factor structure. While it was our intention to make a broadly applicable tool, we acknowledge that this methodological choice may present limitations, as younger and older participants may conceptualise social risks differently.

When translating this scale to younger populations, it is important to incorporate coproduction to enhance our understanding of the lived experience of social risks experienced by those in this younger age bracket. For instance, coproduction approaches, where researchers collaborate directly with adolescents to design and refine the scale, can provide invaluable insights into the unique social challenges faced by this age group.^54^ Additionally, qualitative methods such as focus groups and in-depth interviews can uncover nuanced aspects of social risk that quantitative measures might miss.^54^ Despite these, the PSRS is a valid tool for the empirical assessment of individual differences in social risk perceptions, but ongoing refinement and confirmation of the scales structure is necessary.

Future research should explore these distinct elements of social risk in more diverse and representative samples to enhance the generalisability of the findings. If future research establishes robust psychometric properties of the PSRS in clinical contexts, clinicians may eventually consider using it to better understand social risk perceptions that contribute to maladaptive behaviours or distress. For example, clinicians could use the PSRS to identify individuals with heightened sensitivity to social risks, a feature often linked to conditions such as social anxiety, depression and interpersonal difficulties. This information could guide therapeutic approaches by highlighting specific risk perceptions that contribute to maladaptive behaviours or distress. However, until then, the PSRS should be regarded primarily as a research measure rather than a clinical assessment instrument.

## Conclusion

In conclusion, the PSRS offers a reliable and valid measure to assess perceptions of social risk-taking, providing valuable insights into how individuals perceive and engage in social risk behaviours. The scale demonstrated robust psychometric properties, including strong internal consistency and test–retest reliability. We show that social risk is, in fact, not a uniform construct, but rather comprised of several important components. We validated the PSRS against several related constructs, including rejection sensitivity, depressive symptoms and resistance to peer influence. These significant associations demonstrate the scale’s capability to index several other factors, capturing the complexity of social risk. The scale’s robust psychometric properties and nuanced measurment of perceptions of social risk make it a valuable tool for advancing research and informing interventions aimed at reducing social risks and promoting psychological well-being.

## Supplementary material

10.1136/bmjopen-2024-092107 online supplemental file 1

## Data Availability

Data are available upon reasonable request.
