# Supplementary material for: Perceived Social Risk Scale: development and validation in relation to social status and depression in the UK
Source: BMJ Open. 2025 Oct 9;15(10):e092107. doi: 10.1136/bmjopen-2024-092107 (PMC12516996; doi:10.1136/bmjopen-2024-092107)
Supplement: online supplemental file 1 [file bmjopen-15-10-s001.docx]

**Supplementary material: 1 – The original 36 item table**

| Item | Statement | Factor |
| --- | --- | --- |
| 1 | Voicing an unpopular opinion | 4 |
| 2 | Telling a risky joke | 4 |
| 3 | Wearing clothes that your friends wouldn't approve of | 3 |
| 4 | Missing a popular friends party | 3 |
| 5 | Listening to music that your friends don't like | None* |
| 6 | Defending an unpopular opinion that your friends don't believe in | 4 |
| 7 | Not drinking alcohol at a social event where everyone else is | 3 |
| 8 | Spending the weekend alone despite friends wanting to hang out | 3 |
| 9 | Opting out of a popular social media challenge that everyone is participating in | None* |
| 10 | Not laughing at a group's inside joke that you don't find funny | 3 |
| 11 | Expressing enthusiasm for a hobby that your peers find boring or odd | None* |
| 12 | Skipping a popular movie night to watch a documentary instead | 3 |
| 13 | Choosing a career path that is not well-regarded by your social circle | None* |
| 14 | Admitting you haven't seen a popular TV show that everyone is talking about | 1 |
| 15 | Reading a book at a social event instead of mingling | 3 |
| 16 | Quitting a popular activity because it's no longer enjoyable for you | 1 |
| 17 | Not contributing to a group gift that you find too expensive | 2 |
| 18 | Refusing to spread a rumour, even when all your friends are doing it | 1 |
| 19 | Admitting a mistake in front of peers when you could easily hide it | None* |
| 20 | Asking a question in a large group when everyone else seems to understand | None* |
| 21 | Standing up for someone who is being talked about negatively in their absence | None* |
| 22 | Choosing to stay in on the weekend instead of going out with friends | 3 |
| 23 | Refusing to participate in a prank that you feel is in poor taste | 1 |
| 24 | Bringing up a serious topic of discussion at a light-hearted gathering | 2 |
| 25 | Not gossiping about someone when your friends are too | 1 |
| 26 | Choosing to be the designated driver when everyone else plans to drink | None* |
| 27 | Standing up for a political belief that is unpopular within your friend group | 2 |
| 28 | Admitting you don't know something when asked | None* |
| 29 | Declining to cheat on a test, even when friends assure you it's safe | 1 |
| 30 | Choosing to walk away from a group that is mocking someone else | 1 |
| 31 | Not altering your appearance when it's the norm in your group | 1 |
| 32 | Stating that you don't like a widely loved celebrity | 1 |
| 33 | Refusing to change your opinion just to fit in with a group discussion | 1 |
| 34 | Not using word or phrases that everyone else is using because you don't like it | 1 |
| 35 | Choosing a different meal at a restaurant when all of your friends are having the same | 1 |
| 36 | Not lying to authority figures when your peers are encouraging you to | 1 |

**Table S1**: This table presents the original 36 items used in the Exploratory Factor Analysis (EFA) for the development of the Perceived Social Risk Scale (PSRS). Each item reflects a potential socially risky behaviour, and participants were instructed to rate how much risk they would feel engaging in each behaviour on a sliding scale from "Not at all risky (1)" to "Very risky (7)." The responses were collected online with numerical values visible along the slider. The table includes the statement for each item and its corresponding factor. Factor None refers the 9 items that loaded below 0.40 on any factor, and were thus removed from the final scale. Factor 1 (PSRS) = **Authenticity and integrity**. Factor 2 (PSRS) = **Social assertiveness.** Factor 3 (PSRS) = **Reservedness,** Factor 4 (PSRS) = **Social nonconformity.** Higher scores indicate a greater social risk perception.

|  | **Authenticity**  **& Integrity** | **Social  Assertiveness** | **Reservedness** | **Social Nonconformity** | **MacArthur**  **Scale** | **DOSPERT** | **Dass** | **Belongingness** | **O^2^S^3^** | **RPI** |
| --- | --- | --- | --- | --- | --- | --- | --- | --- | --- | --- |
| **Authenticity & Integrity** | - | **0.51***** | **0.66***** | **0.30 ***** | -0.08 | **-0.16***** | 0.09 | **-0.15***** | 0.07 | -0.04 |
| **Social  Assertiveness** | **0.51***** | - | **0.53***** | **0.49 ***** | -0.06 | **-0.10*** | **0.10*** | **-0.11*** | **0.17***** | -**0.19***** |
| **Reservedness** | **0.66***** | **0.53 ***** | - | **0.46 ***** | -0.00 | -0.08 | 0.05 | -0.06 | **0.20***** | -**0.21***** |
| **Social Nonconformity** | **0.30***** | **0.49***** | **0.46***** | - | **-0.11 *** | -0.05 | **0.12*** | -0.07 | **0.16***** | -0.16*** |
| **MacArthur Scale** | -0.08 | -0.06 | -0.00 | **-0.11 *** | _ | -0.03 | **-0.37***** | **0.28***** | **-0.13*** | 0.03 |
| **DOSPERT** | **-0.16***** | **-0.10*** | -0.08 | -0.05 | -0.03 | _ | 0.00 | -0.09 | -0.04 | **0.24***** |
| **Dass** | 0.09 | **0.10*** | 0.05 | **0.12 *** | **-0.37***** | 0.00 | _ | **-0.64***** | **0.36***** | **-0.16***** |
| **Belongingness** | **-0.15***** | **-0.11*** | -0.06 | -0.07 | **0.28***** | -0.09 | **-0.64***** | _ | -0.01 | -0.16*** |
| **O^2^S^3^** | 0.07 | **0.17***** | 0.20*** | **0.16 ***** | **-0.13*** | -0.04 | **0.36***** | -0.01 | _ | **0.14***** |
| **RPI** | -0.04 | **-0.19***** | -0.21*** | **-0.16 ***** | 0.03 | **0.24***** | **-0.16***** | **-0.16***** | 0.14*** | _ |

**Table S2:** Convergent and Concurrent Validity of the Perceived Social Risk Scale. This table presents the Pearson correlation coefficients between the four subscales of the Perceived Social Risk Scale (PSRS) – Authenticity & Integrity, Social assertiveness, Reservedness, and Social Nonconformity – and various psychological and social measures, including perceived social status (MacArthur Scale), risk-taking propensity (DOSPERT), depressive symptoms (DASS), sense of belonging, online and offline social sensitivity (O^2^S^3^), and resistance to peer influence (RPI). Significant correlations indicate that each subscale is meaningfully related to these constructs, providing evidence for the PSRS’s convergent and concurrent validity. The asterisks denote levels of significance (* p < .05, *** p < .001)

**Supplementary material 2 – Moderation analysis output:**

**2a) Hypothesis 1: Depressive Symptoms Will Moderate the Relationship Between PSRS and Age**

| Predictor | β | SE | t | p |
| --- | --- | --- | --- | --- |
| Intercept | 2.943 | 0.227 | 12.949 | <0.001 *** |
| Age | -0.015 | 0.005 | -2.814 | 0.005 ** |
| Dass | -0.005 | 0.012 | -0.399 | 0.690 |
| Age * Dass | 0.000 | 0.000 | 1.093 | 0.275 |

**Table S2**: This table presents the results of the regression analysis testing Hypothesis 1, which posits that depressive symptoms will moderate the relationship between perceived social risk (PSRS) and age. The predictors include age, depressive symptoms (Dass), and the interaction term between age and depressive symptoms. The coefficients (β), standard errors (SE), t-values (t), and p-values (p) are reported. The results indicate that while age is significantly associated with perceived social risk, depressive symptoms do not significantly moderate this relationship.

**2b) Hypothesis 2a: Local Perceived Social Status (as measured by the MacArthur Scale Local) Will Moderate the Relationship Between PSRS and Depressive Symptoms, While Controlling for Income**

| **Predictor** | **β** | **SE** | **t** | **p** |
| --- | --- | --- | --- | --- |
| Intercept | 2.974 | 0.241 | 12.343 | <0.001 *** |
| Perceived Local Social Status | -0.079 | 0.040 | -2.002 | 0.046 * |
| Dass | -0.019 | 0.012 | -1.625 | 0.105 |
| Income | -0.061 | 0.042 | -1.469 | 0.143 |
| Perceived Local Social Status * Dass + Income | 0.005 | 0.002 | 2.360 | 0.019 * |

**Table S3:** This table presents the results of the regression analysis testing Hypothesis 2a, which examines whether perceived local social status moderates the relationship between perceived social risk (PSRS) and depressive symptoms (Dass), while controlling for income. The predictors include perceived local social status, depressive symptoms, income, and the interaction term between perceived local social status and depressive symptoms. The coefficients (β), standard errors (SE), t-values (t), and p-values (p) are reported. The results indicate that perceived local social status significantly moderates the relationship between PSRS and depressive symptoms, while the direct effects of depressive symptoms and income are not significant.

**H2b) Hypothesis 2b: National Perceived Social Status (as measured by the MacArthur Scale National) Will Moderate the Relationship Between PSRS and Depressive Symptoms, While Controlling for Income**

| **Predictor** | **β** | **SE** | **t** | **p** |
| --- | --- | --- | --- | --- |
| Intercept | 2.684 | 0.221 | 12.132 | <0.001 *** |
| Perceived National Social Status | -0.022 | 0.036 | -0.619 | 0.536 |
| Dass | -0.003 | 0.011 | -0.265 | 0.791 |
| Income | -0.074 | 0.042 | -1.750 | 0.081 . |
| Perceived National Social Status * Dass + Income | 0.002 | 0.002 | 1.068 | 0.286 |

**Table S4**: This table presents the results of the regression analysis testing Hypothesis 2b, which examines whether perceived national social status moderates the relationship between perceived social risk (PSRS) and depressive symptoms (Dass), while controlling for income. The predictors include perceived national social status, depressive symptoms, income, and the interaction term between perceived national social status and depressive symptoms. The coefficients (β), standard errors (SE), t-values (t), and p-values (p) are reported. The results indicate that perceived national social status does not significantly moderate the relationship between PSRS and depressive symptoms, and neither the direct effects of depressive symptoms nor income are significant.

**2c) Hypothesis 3a): A Sense of Belonging Will Moderate the Relationship Between PSRS and Depressive Symptoms**

| **Predictor** | **β** | **SE** | **t** | **p** |
| --- | --- | --- | --- | --- |
| Intercept | 2.779 | 0.260 | 10.688 | <0.001 *** |
| Belonging | -0.081 | 0.050 | -1.619 | 0.106 |
| Dass | 0.001 | 0.011 | 0.064 | 0.949 |
| Belonging * Dass | 0.001 | 0.002 | 0.270 | 0.787 |

**Table S5:** This table presents the results of the regression analysis testing Hypothesis 3a, which posits that a sense of belonging will moderate the relationship between perceived social risk (PSRS) and depressive symptoms (Dass Total). The predictors include sense of belonging, depressive symptoms, and the interaction term between sense of belonging and depressive symptoms. The coefficients (β), standard errors (SE), t-values (t), and p-values (p) are reported. The results indicate that sense of belonging does not significantly moderate the relationship between PSRS and depressive symptoms, and neither the direct effects of sense of belonging nor depressive symptoms are significant.

**Hypothesis 3b) Feelings of Rejection and Exclusion Will Moderate the Relationship Between PSRS and Depressive Symptoms**

| **Predictor** | **β** | **SE** | **t** | **p** |
| --- | --- | --- | --- | --- |
| Intercept | 2.895 | 0.219 | 13.222 | <0.001 *** |
| Rejection and Exclusion | -0.112 | 0.045 | -2.478 | 0.014 * |
| Dass | -0.004 | 0.009 | -0.436 | 0.663 |
| Rejection and Exclusion * Dass | 0.001 | 0.002 | 0.583 | 0.560 |

**Table S6:** This table presents the results of the regression analysis testing Hypothesis 3b, which posits that feelings of rejection and exclusion will moderate the relationship between perceived social risk (PSRS) and depressive symptoms (Dass Total). The predictors include feelings of rejection and exclusion, depressive symptoms, and the interaction term between feelings of rejection and exclusion and depressive symptoms. The coefficients (β), standard errors (SE), t-values (t), and p-values (p) are reported. The results indicate that feelings of rejection and exclusion do not significantly moderate the relationship between PSRS and depressive symptoms. However, the main effect of feelings of rejection and exclusion is significant.

**Supplementary material 3) Visualisations of the PSRS associations with other measures**


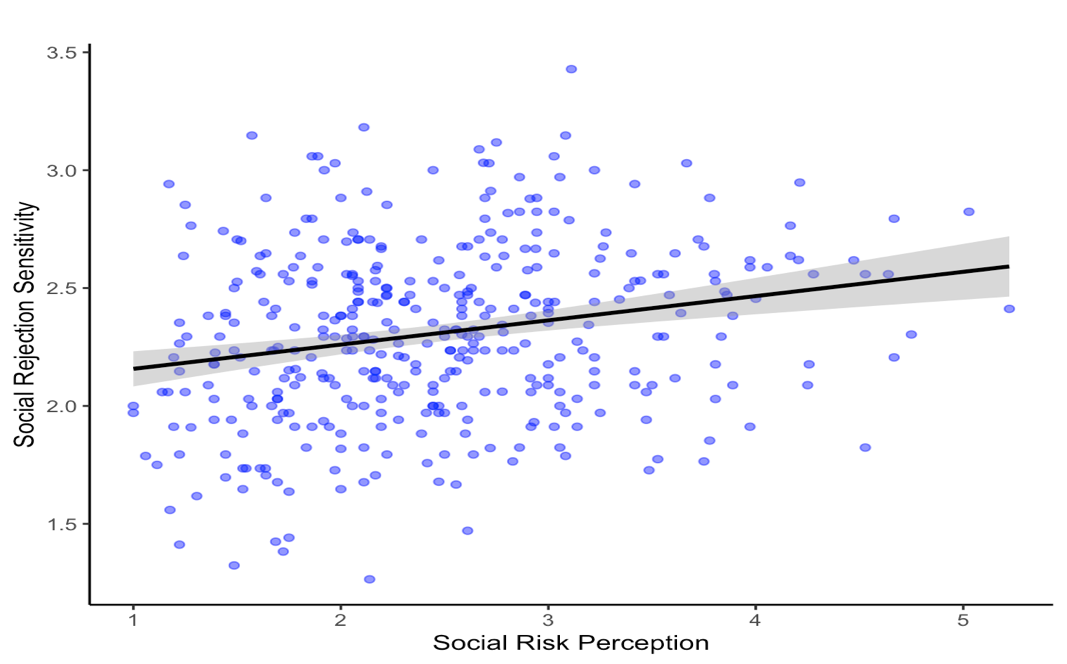


**Figure S1:** The relationship between Social Risk Perception (PSRS) and social rejection sensitivity (O^2^S^3^) is depicted in this scatter plot. Each blue dot represents an individual's score, with social risk perception on the x-axis and social rejection sensitivity on the y-axis. The black trend line, along with its shaded confidence interval, indicates the direction and strength of the relationship. Statistical analysis reveals a significant positive correlation (r(387) = 0.23, p < .001).


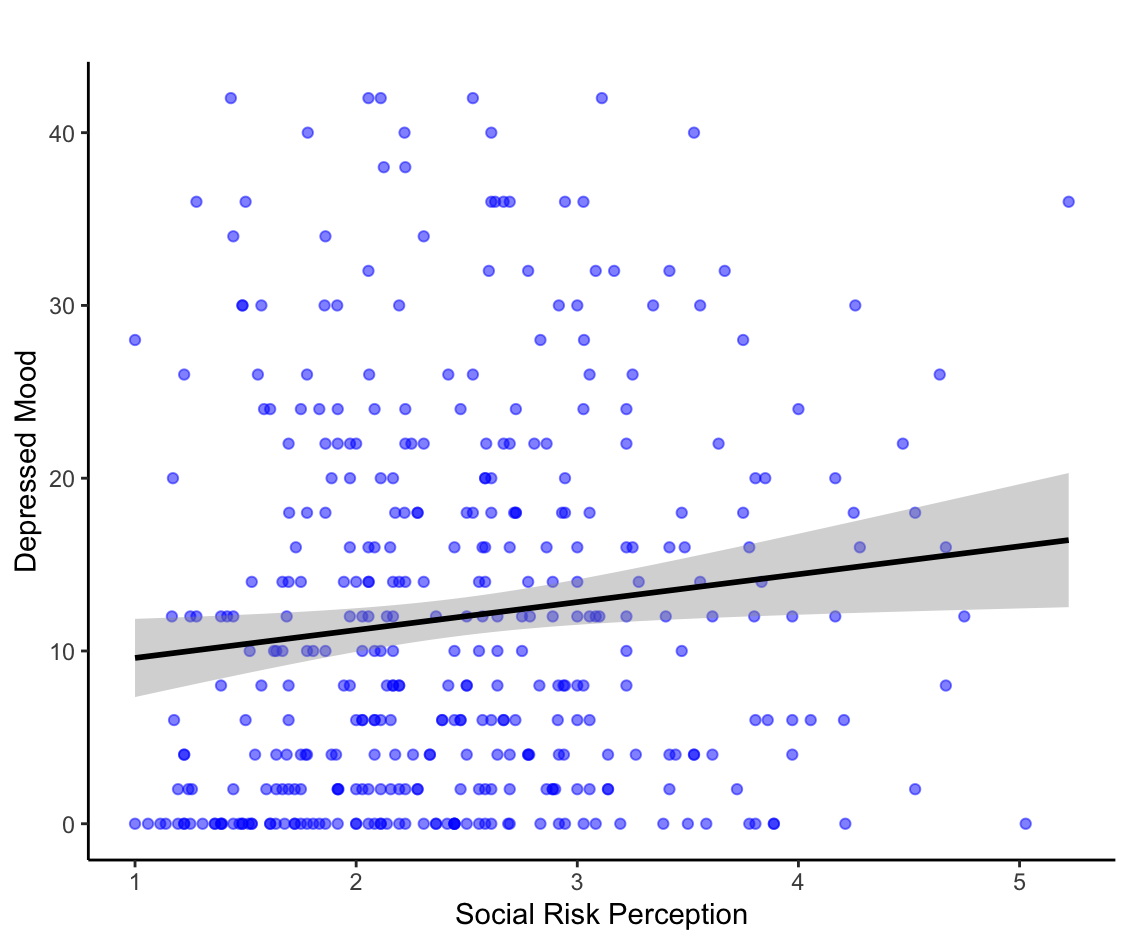


**Figure S2:** The relationship between Social Risk Perception (PSRS) and depressive symptoms (DASS) is depicted in this scatter plot. Each blue dot represents an individual's score, with social risk perception on the x-axis and depressive symptoms on the y-axis. The black trend line, along with its shaded confidence interval, indicates the direction and strength of the relationship. Statistical analysis reveals a significant positive correlation (r(349) = 0.13, p = 0.012).


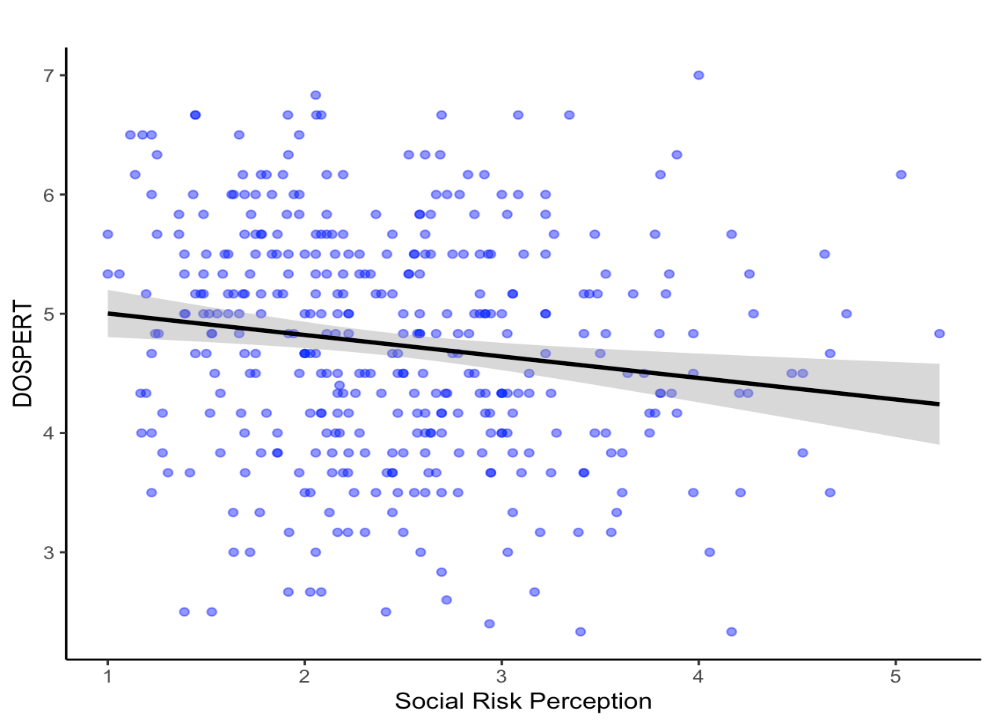


**Figure S3:** The relationship between Social Risk Perception (PSRS) and the likelihood of engaging in social risks (DOSPERT) is depicted in this scatter plot. Each blue dot represents an individual's score, with social risk perception on the x-axis and social risk-taking on the y-axis. The black trend line, along with its shaded confidence interval, indicates the direction and strength of the relationship. Statistical analysis reveals a significant negative correlation *(r(387) = -0.15, p = 0.003).*


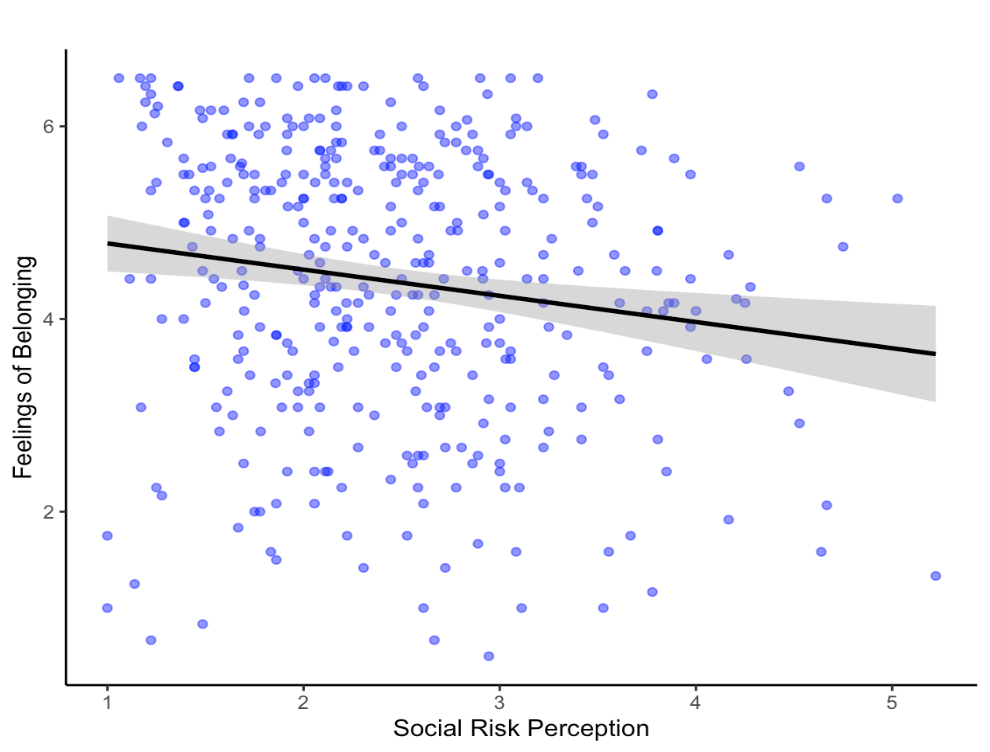


**Figure S4:** The relationship between Social Risk Perception (PSRS) and belongingness (GBS) is depicted in this scatter plot. Each blue dot represents an individual's score, with social risk perception on the x-axis and sense of belonging on the y-axis. The black trend line, along with its shaded confidence interval, indicates the direction and strength of the relationship. Statistical analysis reveals a significant negative correlation *(r(386) = -0.15, p = 0.002).*


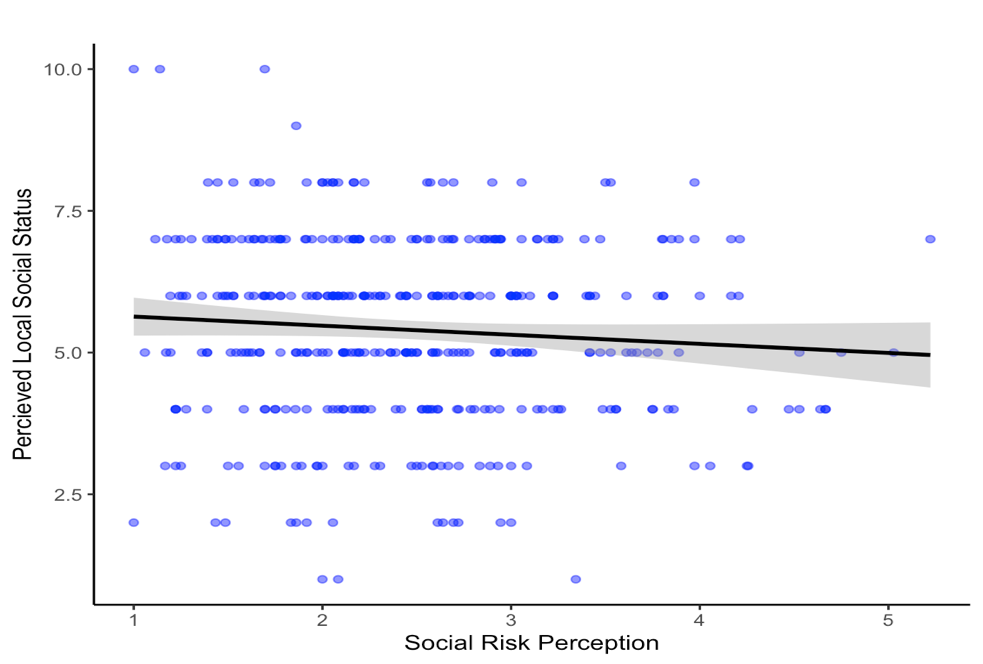


**Figure S5:** The relationship between Social Risk Perception (PSRS) and subjective local social status (MacArthur Local) is depicted in this scatter plots. Each blue dot represents an individual's score, with social risk perception on the x-axis and subjective local social status on the y-axis. The black trend lines, along with their shaded confidence intervals, indicate the direction and strength of the relationships. Statistical analysis reveals negative but not statistically significant correlations between PSRS and MacArthur Local scale *(r(386) = -0.08, p = 0.116)*


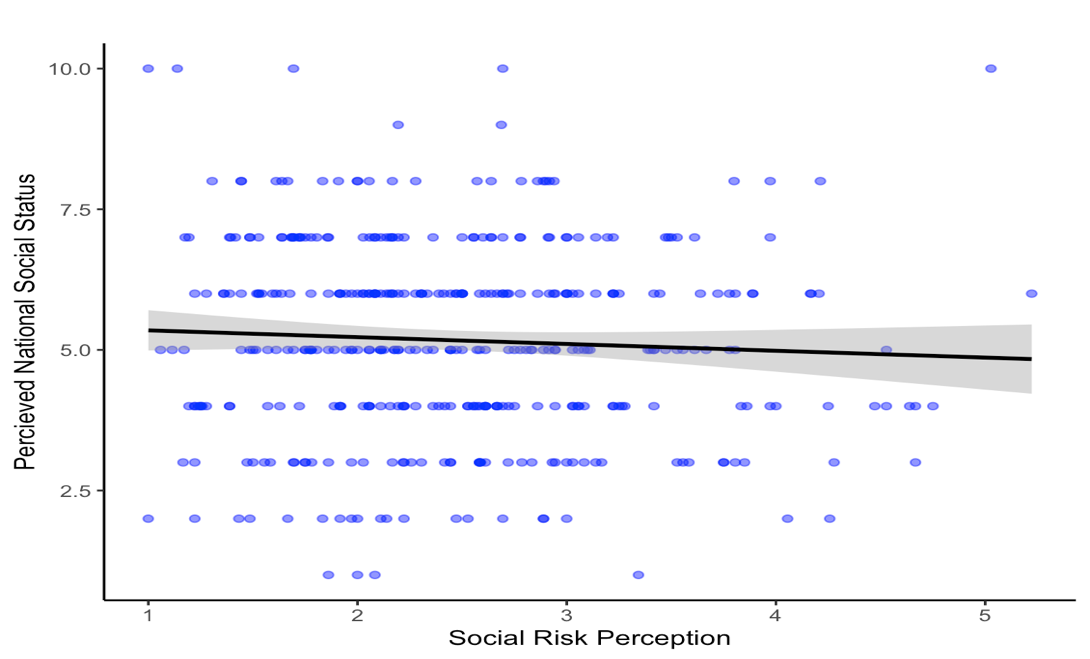


**Figure S6:** The relationship between Social Risk Perception (PSRS) and subjective National social status (MacArthur National) is depicted in this scatter plots. Each blue dot represents an individual's score, with social risk perception on the x-axis and subjective local social status on the y-axis. The black trend lines, along with their shaded confidence intervals, indicate the direction and strength of the relationships. Statistical analysis reveals negative but not statistically significant correlations between PSRS and MacArthur National Scale *(r(386) = -0.06, p = 0.268).*


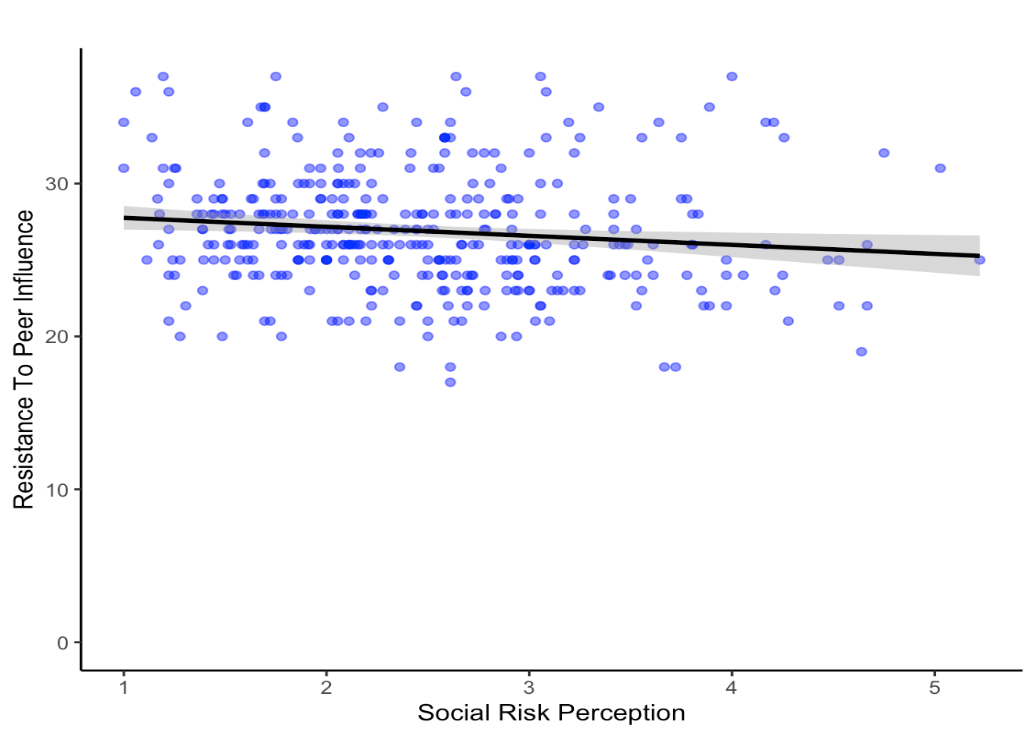


**Figure S7:** The relationship between Social Risk Perception (PSRS) and resistance to peer influence (RPI) is depicted in this scatter plot. Each blue dot represents an individual's score, with social risk perception on the x-axis and resistance to peer influence on the y-axis. The black trend line, along with its shaded confidence interval, indicates the direction and strength of the relationship. Statistical analysis reveals a significant negative correlation *(r(387) = -0.13, p = 0.013).*


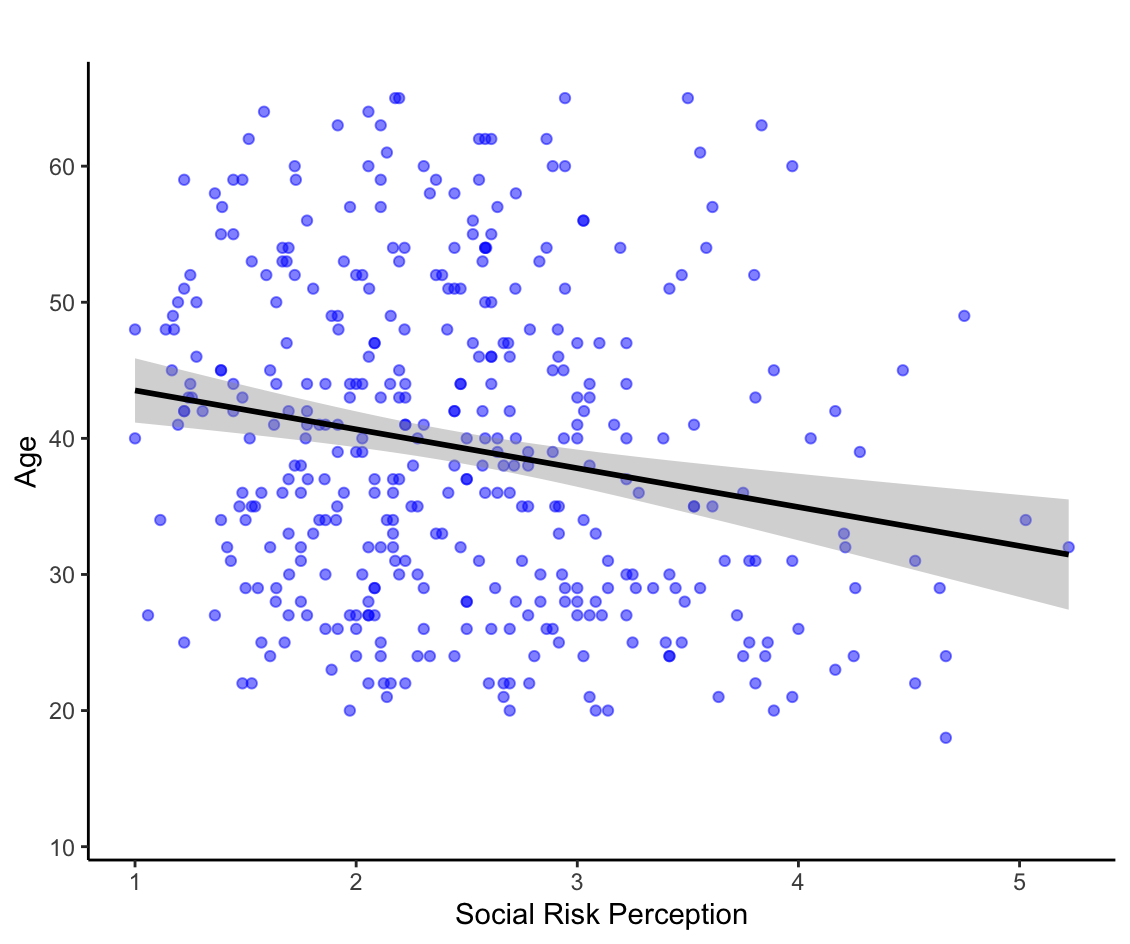


**Figure S8:** The relationship between Social Risk Perception (PSRS) and age is depicted in this scatter plot. Each blue dot represents an individual's score, with social risk perception on the x-axis and age on the y-axis. The black trend line, along with its shaded confidence interval, indicates the direction and strength of the relationship. Statistical analysis reveals a significant negative correlation *(r(387) = -0.20, p < .001).*

**Supplementary material 4) Test-retest of individual factors (13-15 days apart)**

**4) Test-retest of individual factors**

The reliability for Factor 1 ***(Authenticity and Integrity***) was also good, *r(107) = 0.60, p<.001 (95% CI [0.467, 0.710])*. Factor 2 **(Social assertiveness)** also showed good reliability, *r(107) = 0.63, p<.001 (95% CI [0.499, 0.730]).* Factor 3 **(Reservedness)**, however, showed lower reliability, *r(107) = 0.20, p = 0.014, (95% CI [0.014, 0.375]).* Factor 4 **(Social Nonconformity)**, showed good reliability, *r(107) = 0.64, p<.001 (95% CI [0.515, 0.740]*
