# Supplementary material for: Perceived Social Risk Scale: development and validation in relation to social status and depression in the UK
Source: BMJ Open. 2025 Oct 9;15(10):e092107. doi: 10.1136/bmjopen-2024-092107 (PMC12516996; doi:10.1136/bmjopen-2024-092107)
Supplement: Supplementary file 2 [file bmjopen-2024-092107.reviewer_comments.pdf]

# PEER REVIEW HISTORY

BMJ Open publishes all reviews undertaken for accepted manuscripts. Reviewers are asked to complete a checklist review form and are provided with free text boxes to elaborate on their assessment. These free text comments are reproduced below.

**ARTICLE DETAILS**

**Title (Provisional)**

The Perceived Social Risk Scale: Development and Validation in Relation to Social Status and Depression in the UK

**Authors**

Newton, Joseph; Andrews, Jack L.

---

## VERSION 1 - REVIEW

---

| | |
|---|---|
| **Reviewer** | **1** |
| **Name** | **Van Den Bos, Wouter** |
| **Affiliation** | |
| **Date** | **30-Sep-2024** |
| **COI** | **None** |

---

I loved reading this paper, it is about an important topic and introduces a measure that is needed by the scientific community more broadly. However, I did have some reservations after reading it..

The introduction is clear and well structured. However, I have to say it is very self-referential, there are other studies on social influence in adolescence, also on risk taking behavior. From multiple labs and from the past decades, so it would be nice to be a bit more fair and balanced in the review of the literature. Currently there is only one reference outside the Blakemore lab in the first part.

What is also missing is the theory and rationale about the different types of questions and expected context difference one may expect. Why would there be different factors, what might these a priori be, and why? Building of the DOSPERT, there it is very clear there is a difference between gambling and making a friend and that people may differ in their risk perception and willingness. Did the factor structure conform with expectations? We don’t know. This seems more relevant and interesting and to precede how it correlates with all kinds of other measures.

In the methods the authors claim “We then supplemented these items by comprehensively reviewing the theoretical and empirical literature on social risk and related constructs,

ensuring a broad conceptual foundation for item generation." But this is not shared with reader, unfortunately.

Is there any previous evidence for the social risk hypothesis for depression? The way it is presented it follows the early evolutionary psychology logic that just everything must be in some way adaptive, simply because it would not evolve otherwise. It is inconsistent with the one study from Wang that you mention, but are there many others that have supported it over the past two decades?

The status of this hypothesis is not very clear at the moment. From the paragraph it reads as pretty weak and could go both ways , but in results you state "confirming that individuals who scored highly on perceptions of social risk also reported higher levels of depressive symptoms." Hypothesis in pre-reg also does not point in one direction or other. How important is this link for the validation of the questionnaire.

And how does it fit with the finding that sense of belonging does not significantly moderate the relationship between PSRS and depressive symptoms?

In the intro, but also later on, higher self-esteem ,perceived social status and hierarchical position are all heaped together but these are quite distinct concepts. Could the authors be a bit more specific here.

Methods

Isn't it problematic that the factor structure is determined by the young students and than later confirmed in a sample with older participants? Also do you think it is the risk perception that is changing with age or the actual risk (or both). And this also raised the question, why not simply asked for willingness to engage in these risky behaviors, is that not what we are most interested in. Like the DOSPERT has? Note also that in the proposed link between depression and risk taking, it is about risk taking not risk perception that changes. The lack of such measure seems quite a shame. Given that prolific is quite easy and low cost, would the authors consider adding a sample with an additional willingness to engage question like you have in DOSPERT?

We have experienced quite some differences in way of responding between "community" sample students and prolific users. Is there a way to check that in your data, for instance comparing the participants that overlap in age, and see how they score similarly or differently?

The factors

I feel the factors are still quite similar and they are also quite highly correlated. So not yet totally convinced by the need of these four factors. Social Nonconformity is a bit of a misnomer, all examples are examples of non-conformity. Not conforming is the overall risk you are taking if you are taking social risks.

For example, Confidently standing up for beliefs in social situation and "Defending an unpopular opinion that your friends don't believe in" also go really well together but are different factors.

The following statements also are part of two different factors but I think very few people would guess that or correctly which is part of which:

"Defending an unpopular opinion that your friends don't believe in."

"Standing up for a political belief that is unpopular within your friend group"

"Voicing an unpopular opinion"

It may have been helpful to have a prior predictions about the correlations of each scale with different constructs, not just post hoc.

why not ICC for test retest reliability?

It would be good to mention to what extent you consider the effect sizes from all the validity correlations, some are quite low. Not sure if that is problematic from a validation standpoint, now it seems to focus on p-values, but maybe you can give the reader a better sense of the strength of these correlations and what could be expected.

Some thoughts on the item structure

Without the statistics I would say there are activities you do solo (watch a series) but do it bc everybody does it, there are social gatherings you may or may not attend (party), there are joint activities you may opt out of (joint gift), and there are differences in opinion you may or may not voice. These seem all different kinds of context. But the current factors are mixed bags that are not very intuitive. I could also expect differences in action vs inaction, but don't really see that either. Not sure if this is useful. But maybe the authors can spend a little more time reflecting on these factors

Finally, the expectation is built that adolescents will be studied. It is clear that this is an important topic for this age group and there are quite some references to this group, but then it is not studied. Why not?

Also we indeed have a lot of evidence that there are changes in social risk between 10-20 maybe 10-25 but this is not yield the expectation that there would be an age effect between 18-65. There is actually very little literature on this. So I would not present your age finding as something expected but more as something interesting and novel.

---

| | |
|---|---|
| **Reviewer** | **2** |
| **Name** | **Gibson, Melanie** |
| **Affiliation** | **Wellington Regional Hospital** |
| **Date** | **10-Oct-2024** |

**COI** **None**

---

Thank you for inviting me to review manuscript bmjopen-2024-092107 entitled "The development of the perceived social risk scale and its association with social status and depression". Strengths of the study include the large sample and use of a range of scales measuring different constructs. The main weaknesses are the lack of a sampling strategy and the lack of a description of the study sample.

Abstract

Can the authors please add some numbers in the results section to support the statements made.

Introduction

Could the authors please add more detailed description of the anticipated applications of such a tool? While broad suggestions of clinical and research potential are made, it is still unclear how the new tool may be used, particularly clinically.

Could the authors please describe how they ensured that “the items included in our scale reflect everyday social scenarios that people from diverse backgrounds can understand and evaluate”? This step of the research isn’t covered in the methods or results.

Methods

In several places in the manuscript it is stated that people from diverse backgrounds were recruited, yet no sampling strategy is described and no information confirming the diversity (or even defining what diversity is meant here). The lack of a sampling strategy is a major limitation, raises questions of regarding bias, and means that disaggregation of results by characteristics such as sex is probably not possible. The lack of a description of the study sample means it is very unclear who was in the sample and who the developed tool applies to.

Could the authors describe how the DASS short version was chosen out of the many short form screening tools for Depression?

Could the authors please describe the rationale for conducting the EFA in a sample of 18-25 year olds and then the CFA in a wider age group (18-65 year olds)? Are there any potential issues with this difference in samples?

Results

Could the authors give an age breakdown for the sample involved in CFA?

Could the authors also give the age and sex breakdown for the sample who took part in the test-retest reliability phase?

Discussion and Conclusion

It is concluded that the “Perceived Social Risk Scale (PSRS) offers a reliable and valid measure to assess perceptions of social risk-taking” but without a representative sample of the population, and without a description of the study sample, we don’t know who this measure is reliable or valid for.

---

## VERSION 1 - AUTHOR RESPONSE

**Reviewer: 1**

**Wouter Van Den Bos**

Comments to the Author:

I loved reading this paper, it is about an important topic and introduces a measure that is needed by the scientific community more broadly. However, I did have some reservations after reading it..

We thank Professor Van Den Bos for his positive appraisal of our paper, and for the detailed comments which have been incredibly helpful in refining and improving our paper.

1. The introduction is clear and well structured. However, I have to say it is very self-referential, there are other studies on social influence in adolescence, also on risk taking behavior. From multiple labs and from the past decades, so it would be nice to be a bit more fair and balanced in the review of the literature. Currently there is only one reference outside the Blakemore lab in the first part.

We appreciate this comment and have now balanced the introduction out by incorporating a wider literature on adolescence, risk-taking and social influence from other labs. Specifically, Chein et al., 2011; Prinstein & Dodge, 2008; Albert & Steinberg; 2008; Steinberg 2008.

Additions made:

**“This is surprising given that health and legal risk taking often occurs in the presence of others and is heavily influenced by social norms[3]. “**

**“Longitudinal studies have demonstrated that susceptibility to peer influence decreases with age as self-regulation and autonomy develop[4] .”**

**“This is supported by other studies which have consistently shown that adolescents are more likely to engage in risky behaviours when peers are present, highlighting the importance of peer dynamics in decision-making[6 7].”**

2. What is also missing is the theory and rationale about the different types of questions and expected context difference one may expect. Why would there be different factors, what might these a priori be, and why? Building of the DOSPERT, there it is very clear there is a difference between gambling and making a friend and that people may differ in their risk perception and willingness. Did the factor structure conform with expectations? We don’t know. This seems more relevant and interesting and to precede how it correlates with all kinds of other measures.

Thank you for this comment. We have now included more detail that explains the theory as to why social risk would be a multi-dimensional construct. Building off the DOSPERT, we discuss other important constructs that we considered:

**Included in Introduction:**

**”In addition, existing work has characterised and measured social risk-taking as a uniform, domain general construct[1 30]. However, there is reason to hypothesise that social risk taking is multifaceted, comprised of various sub domains. For example, social risks that involve approach behaviours (e.g., standing up for an unpopular peer) could be considered conceptually distinct from social risks that involve avoidance behaviours (e.g., missing a popular friend’s party), which too could be considered distinct from social risks that violate norms (e.g., reading a book during a party).**

**As such, we can consider social risk as being comprised of multiple domains. *Approach-related* social risks, such as standing up for an unpopular peer or expressing an unpopular opinion, involve confronting group dynamics and prioritising personal authenticity, at the expense of risking social harmony. *Avoidance-related* social**

**risks, such as declining a social invitation or missing a popular event, involve decisions that limit opportunities for social connection and inclusion but serve to protect individual boundaries or preferences. Conversely, social risks which could be considered *norm-violations*, such as stating that you don't like a widely loved celebrity or wearing clothes that your friends wouldn't approve of, involve intentional divergence from group expectations, risking disapproval but asserting individuality. These distinctions highlight a multi-dimensionality of social risks that prior social risk measurement hadn't covered."**

**Included in Discussion:**

**"Whilst our results supported a multidimensional account of social risk which broadly aligned with our predictions, our findings indicated that certain factors were more nuanced than we anticipated. For example, rather than a single factor capturing norm-violating behaviours, our results suggested a meaningful distinction between social assertiveness, which involves confidently advocating for personal beliefs in social settings, and nonconformity, which captures risks associated with behaviours that diverge from group norms. This suggests that while both assertiveness and nonconformity involve some degree of standing out from the group, they may be distinct in how individuals perceive the social consequences of these actions."**

3. In the methods the authors claim "We then supplemented these items by comprehensively reviewing the theoretical and empirical literature on social risk and related constructs, ensuring a broad conceptual foundation for item generation." But this is not shared with reader, unfortunately.

We have now added in more detail to this section:

**"We reviewed each social risk item in both scales, and hypothesised that there would be different underlying dimensions between some questions on both scales (e.g., approach versus avoidance behaviours). We then supplemented these items by comprehensively reviewing the theoretical and empirical literature on social risk and related constructs included examining research and measures related to belongingness[38 34], rejection sensitivity[31 39], and resistance to peer influence[33], ensuring a broad conceptual**

**foundation for item generation. Items were constructed to reflect a wide array of social risk behaviours that reflect different types of social risk. Finally, items were carefully crafted using clear and accessible language, avoiding jargon or culturally specific references. This approach prioritised scenarios that were broadly relatable, such as "Not laughing at a group's inside joke that you don't find funny," rather than situations tied to specific cultural norms."**

We have also added in some text to clarify the differences between them:

**"For instance, social risk items included statements such as: "Telling a risky joke" (approach-related social risk), "Wearing clothes that your friends wouldn't approve of" (norm-violating social risk), "Not contributing to a group gift that you find too expensive" (avoidance-related social risk), and "Refusing to spread a rumour, even when all your friends are doing it" (norm-violating social risk). "**

4. Is there any previous evidence for the social risk hypothesis for depression? The way it is presented it follows the early evolutionary psychology logic that just everything must be in some way adaptive, simply because it would not evolve otherwise. It is inconsistent with the one study from Wang that you mention, but are there many others that have supported it over the past two decades?

We have now added in more detail here (Ren et al., 2015; Elmer & Stadtfeld, 2020, Girard et al., 2013, Beck & Alford, 2009) and have provided more evidence for the social risk hypothesis of depression:

**"Empirical evidence provides compelling support for this hypothesis. It is well established that depressive symptoms profoundly affect social behaviour. For example, it has been found that university students with higher depressive symptoms engaged less frequently in social interactions, preferred one-on-one (dyadic) engagements over group settings, and often interacted with others who also exhibited depressive symptoms[12]. Such behaviours could serve to limit negative social evaluations, aligning with the hypothesis that depression-related behaviours aim to mitigate social risks. It has been further**

**demonstrated that individuals that had experienced social exclusion showed a preference for solitude[13]. This behaviour can be interpreted as a self-protective strategy to reduce exposure to further social threats, consistent with the adaptive function proposed by the Social Risk Hypothesis. In addition, there is evidence that low mood can increase affective theory of mind, suggesting that depressive symptoms may increase orientation towards social information and increase social sensitivity[14]. This is consistent with the social risk hypothesis, as increased orientation to social cues may improve emotion detection and therefore one's ability to navigate unstable social situations[15].”**

&

**“It is also important to consider that social withdrawal, a common symptom associated with depressed mood, could be attributed to factors beyond heightened perceived social risk. For instance, reduced energy levels and anhedonia—core symptoms of depression—might inherently limit social engagement[17] due to diminished motivation and pleasure which impair the ability to sustain relationships. This raises questions about whether social withdrawal reflects an adaptive mechanism or simply the functional outcomes of these symptoms. Therefore, further work to understand how the association between social risk perception and depression is influenced by social standing is crucial for disentangling these findings.”**

5. The status of this hypothesis is not very clear at the moment. From the paragraph it reads as pretty weak and could go both ways , but in results you state "confirming that individuals who scored highly on perceptions of social risk also reported higher levels of depressive symptoms." Hypothesis in pre-reg also does not point in one direction or other. How important is this link for the validation of the questionnaire.

**Information that we have added to the introduction around our hypothesis:**

**“Informed by theoretical work suggesting that social risk is multifaceted, we anticipated that the PSRS would comprise multiple factors. We first ran an exploratory factor analysis (EFA), and consistent with our expectations, the EFA demonstrated that social risk comprised of four distinct but related dimensions (see results below). Following this, we**

pre-registered a number of subsequent hypotheses. We first hypothesised that the four-factor structure would be confirmed through confirmatory factor analysis (CFA). We also hypothesised that this four-factor solution would provide a better fit than a one-factor model. Second, we hypothesised that the PSRS would exhibit good concurrent validity by correlating with the social risk sub-scale of the DOSPERT[30] and the Online and Offline Social Sensitivity Scale ($O^2S^3$) [31]. Third, we hypothesised the PSRS to demonstrate good convergent validity with depressive symptoms (measured by the DASS-7) [32], the Resistance to Peer Influence Scale[33], perceived social status[21], and belonging[34]. Finally, we hypothesised that PSRS scores would negatively correlate with age, such that older individuals would report lower social risks perception than younger individuals.

Additionally, we hypothesised that depressive symptoms would moderate the relationship between perceived social risk and age, with those experiencing higher depressive symptoms perceiving greater social risk across all ages. We also hypothesised that perceived social status would moderate the relationship between perceived social risk and depression, with a stronger relationship for individuals lower in objective income. Lastly, we hypothesised that belonging would moderate the relationship between perceived social risk and depression, with weaker associations among those reporting greater feelings of belonging."

**Information that we have added into our discussion as to why belonging did not moderate the relationship between the PSRS and depressive symptoms.**

"Counter to our hypothesis, we found that a general sense of belonging did not significantly moderate the relationship between PSRS and depressive symptoms. One possible explanation for our finding that a general sense of belonging did not impact the relationship between social risk perception and depression, is that an individual may feel generally included in their social environment yet still perceive particular social actions as risky. This suggests that belonging alone may be insufficient to mitigate the distress associated with high social risk perception, underscoring the need to consider other factors when examining how social risk and depression intersect."

In the intro, but also later on, higher self-esteem ,perceived social status and hierarchical position are all heaped together but these are quite distinct concepts. Could the authors be a bit more specific here.

We thank the reviewer for pointing this out. We have been more specific here:

**“Self-esteem, perceived social status, and hierarchical position are distinct, yet interrelated constructs that influence social behaviour and mental health outcomes. Self-esteem refers to an individual's subjective evaluation of their own worth, encompassing feelings of competence and personal value[18]. In contrast, perceived social status reflects one's self-assessed rank within a social hierarchy, often influenced by comparisons with peers and societal norms[19]. Hierarchical position, however, denotes an objective placement within a structured system, such as organisational roles or dominance hierarchies, independent of personal evaluations[20].“**

We included perceived social status in this research as it most directly captures how individuals view themselves in relation to their immediate social environment. While self-esteem and hierarchical position are important, they do not fully capture the subjective, context-specific sense of rank that individuals internalise when making socially risky decisions. Perceived social status, by contrast, is sensitive to local comparisons, peer influence, and everyday status-related concerns, all highly relevant to understanding social risk perception and its impact on mental health.

**Methods**

6. Isn’t it problematic that the factor structure is determined by the young students and than later confirmed in a sample with older participants? Also do you think it is the risk perception that is changing with age or the actual risk (or both). And this also raised the question, why not simply asked for willingness to engage in these risky behaviors, is that not what we are most interested in. Like the DOSPERT has? Note also that in the proposed link

between depression and risk taking, it is about risk taking not risk perception that changes. The lack of such measure seems quite a shame.

We thank the reviewer for the comment here. There are many cases where questionnaires are developed in one sample and extended to broader populations at the CFA stage. Our results are reassuring given the good fit indices we find in both the EFA and CFA sample, suggesting that the constructs remain consistent across age groups. Indeed, the CFA sample does also capture the full age range of the EFA sample but extends this towards older adults.

Thank you for raising the question around risk perception vs actual risk. This is an important line of enquiry that we hope to follow up on in a subsequent study. We acknowledge our lack of data on this and have listed this in our limitations section. We are unfortunately unable to collect new data as part of this project, but plan to extend these findings in future work to examine how our scale index's self-reported real-world risk taking. In terms of the link between depression and risk taking, we examined perceptions in our sample as previous work has shown a relationship between risk perception (on the social risk sub scale of the HSRQ) and depression. That is, higher social risk perception is related to greater depressive symptomatology.

**Added to limitations:**

**"Another point of difference between the PSRS and the DOSPERT[30] is that the focus of the PSRS is on risk perception rather than a self-reported willingness to engage in social risk-taking behaviours. While perceived risk and willingness to engage in risk-taking behaviours are conceptually related, they are distinct constructs, and it remains unclear whether differences in perceived social risk translate into differences in behavioural risk-taking. The absence of a direct measure of willingness to engage in social risks in this study limits our ability to draw conclusions about behavioural outcomes, particularly given the proposed links between depressive symptoms and actual risk-taking behaviours."**

7. Given that prolific is quite easy and low cost, would the authors consider adding a sample with an additional willingness to engage question like you have in DOSPERT?

We have experienced quite some differences in way of responding between “community” sample students and prolific users. Is there a way to check that in your data, for instance comparing the participants that overlap in age, and see how they score similarly or differently?

We thank the reviewer for raising this point. We have now ICC tested based on age across our EFA and CFA sample, and show excellent consistency across samples dependent upon age. We agree adding another sample with a willingness to engage measure would be an important line of enquiry for future work, however, we are not in a position to collect new data for this current project. We have however, ICC tested the EFA (university sample) and the CFA (Prolific) by age, and we show that the ICC comes out at 0.951.

**“Cross Sample Consistency**

**We then compared responses across participants from the EFA and CFA based on age. Specifically, to ensure consistency between our University sample and our Prolific we tested how participants with the same age (18-25) responded. The results indicated excellent consistency between University and Prolific sampling based on age, *ICC = 0.95, (95% CI: [0.944, 0.957]). “***

**The factors**

8. I feel the factors are still quite similar and they are also quite highly correlated. So not yet totally convinced by the need of these four factors. Social Nonconformity is a bit of a misnomer, all examples are examples of non-conformity. Not conforming is the overall risk you are taking if you are taking social risks.

For example, Confidently standing up for beliefs in social situation and "Defending an unpopular opinion that your friends don't believe in” also go really well together but are different factors.

The following statements also are part of two different factors but I think very few people would guess that or correctly which is part of which:

"Defending an unpopular opinion that your friends don't believe in."
"Standing up for a political belief that is unpopular within your friend group"
"Voicing an unpopular opinion" It may have been helpful to have a prior predictions about the correlations of each scale with different constructs, not just post hoc.

We appreciate the reviewer's concern regarding the apparent overlap among the four factors, as well as the label "Social Nonconformity." While we acknowledge that these subscales are correlated, our analyses showed that a four-factor model best represented the data compared to a single-factor solution. Although all items involve some degree of not conforming to the group, the patterns of loadings suggest that people differentiate between distinct types of social risks. Specifically, "Social Assertiveness" reflects an active, vocal stance, whereas "Social Nonconformity" captures departures from group norms, such as opting out of group activities. Moreover, although these factors overlap, we allowed for this in our EFA model, given it is unlikely we would find factors that are unrelated. In addition, our approach is data drive, such that we are guided by the EFA findings, rather than categorising the items based on our own conceptualisations.

With that in mind, we recommend two approaches when using our scale. First, researchers seeking an overall sense of how socially risky a person finds various behaviours can use the total PSRS score. Second, when there is theoretical or practical interest in examining subtler differences, researchers can focus on the subscales. This approach mirrors the use of other multidimensional measures (e.g., personality inventories) that maintain a single overarching construct while offering more detailed subcategories for specific analyses.

Finally, we agree that it would have been helpful to state a priori hypotheses about how each subscale should correlate with external criteria (e.g., depressive symptoms). Our current findings provide preliminary evidence that individuals do make meaningful distinctions among different forms of social risk, even if all share the theme of resisting group pressures. We encourage future research to clarify whether particular subscales independently predict distinct behavioural or psychological outcomes.

9. why not ICC for test retest reliability?

We thank the reviewer for this comment. We have ICC tested, and that is now included in the paper. The results are shown above in point 6.

10. It would be good to mention to what extent you consider the effect sizes from all the validity correlations, some are quite low. Not sure if that is problematic from a validation standpoint, now it seems to focus on p-values, but maybe you can give the reader a better sense of the strength of these correlations and what could be expected.

We thank the reviewer for these comments. We have added into the limitations our thoughts on the strength of the effect sizes:

**"In addition, while the correlations between the PSRS and related constructs were statistically significant, the strength of these associations ranged from small to moderate. These effect sizes are consistent with theoretical expectations, as social risk perception is likely influenced by a multitude of factors beyond the constructs examined in this study, such as further individual differences (e.g., neuroticism), the cultural context (e.g., collectivistic vs. individualistic norms), prior experiences of social acceptance or rejection, situational aspects of the peer group, and any specific mental health conditions (such as mood disorders). While these findings provide preliminary evidence of the PSRS's validity, it highlights the need for further research to explore additional predictors and outcomes of social risk perception."**

**Some thoughts on the item structure**

11. Without the statistics I would say there are activities you do solo (watch a series) but do it bc everybody does it, there are social gatherings you may or may not attend (party), there are joint activities you may opt out of (joint gift), and there are differences in opinion you may or may not voice. These seem all different kinds of context. But the current factors are mixed bags that are not very intuitive. I could also expect differences in action vs inaction, but don't really see that either. Not sure if this is useful. But maybe the authors can spend a little more time reflecting on these factors

We appreciate the reviewer's thoughtful insights into the potential distinctions between solo and group activities, as well as action versus inaction in social risk-taking. While the current factors reflect the results of data-driven analyses, we agree that these distinctions may represent important theoretical categories that are not fully captured in the current structure. In the revised manuscript, we have added a discussion of these points and proposed future research to explore whether these distinctions could enhance the PSRS's conceptual clarity and practical utility:

**"It is also important to consider that while the factors derived from our analysis provide insight into the dimensions of social risk, we acknowledge that they may appear less intuitive due to overlaps in context (e.g., solo vs social activities) or action type (e.g., action vs inaction). Future research could further refine these factors by exploring context-specific distinctions or alternative theoretical frameworks, such as the role of action vs inaction in shaping responses to social risk-taking."**

12. Finally, the expectation is built that adolescents will be studied. It is clear that this is an important topic for this age group and there are quite some references to this group, but then it is not studied. Why not?

We have added more information on our focus on the adult sample. Our intentions were to develop a measure that appropriately captured social risk behaviours among older adolescents (18+) and adults as this period of development is often overlooked when examining age related change in concepts traditionally thought of as typically adolescent. We did not plan to create a one size fits all measure for younger adolescents and adults as the types of social risks they experience are likely qualitatively different to those of adults. Therefore, we would have expected to ask different questions to a younger age group. As noted, there is already a good body of work examining social risk in adolescence, and our intentions were to develop a measure that can advance this work into older samples.

Added to the introduction:

**“However, research on social risk-taking has primarily focused on adolescence, and it is not fully understood how social risk perception functions across adulthood. In this paper, we aimed to develop and validate the Perceived Social Risk Scale (PSRS), a novel tool designed to measure perceived social risk-taking. We further aim to validate and test perceptions of social risk, with a number of other measures, and then assess the relationship between depressive symptoms and perceived social status. We aimed to validate this scale for older adolescents and adults (18 – 65). By designing the PSRS for individuals aged 18 to 65, we are able to gain greater understanding of age-related changes in social risk perception beyond adolescence.”**

**“Our scale aims to dissect social risk into specific sub-categories, allowing us to make more precise predictions about social risk behaviours. Previous scales have not offered the granularity to draw such distinctions, which is a gap that our scale addresses. Finally, by creating a measure that acutely captures social risk perceptions among older adolescents and adults we will be better able to design studies to assess developmental trajectories of social risk-taking across the lifespan.”**

13. Also we indeed have a lot of evidence that there are changes in social risk between 10-20 maybe 10-25 but this is not yield the expectation that there would be an age effect between 18-65. There is actually very little literature on this. So I would not present your age finding as something expected but more as something interesting and novel.

We appreciate this framing of our findings as novel and interesting. We have now emphasised this in the paper:

**“Most existing research on developmental shifts in social risk-taking has concentrated on adolescence to early adulthood (ages 10–24), leaving little known about how social risk perception evolves in later adult years. Consequently, our finding that perceived social risk declines across the 18–65 age range offers a novel insight into an underexplored period of development. These results warrant further investigation into**

**how factors such as shifting life roles, accumulating social experiences (such as rejection), or changing social priorities influence social risk perception into mid- and late-adulthood."**

**Reviewer: 2**

Dr. Melanie Gibson, Wellington Regional Hospital

We thank Dr Gibson for her comments about our paper. The suggestions and comments have been very useful in further clarifying the usage of the PSRS.

**Abstract**

14. Can the authors please add some numbers in the results section to support the statements made.

We have added some statistics to our abstract:

**" The PSRS showed strong internal consistency *($\alpha$ = .96)* and good test-retest reliability *(ICC = 0.70)*. Perceptions of social risks significantly declined with age *(r = -0.20, p < .001)*, and factor analyses confirmed that the PSRS differentiates among four distinct but related social risk constructs: authenticity and integrity ($\alpha$ = .91), social assertiveness ($\alpha$ = .72), reservedness ($\alpha$ = .83), and social nonconformity ($\alpha$ = .72). Higher PSRS scores were associated with increased sensitivity to social rejection *(r = 0.23, p < .001)*, elevated depressive symptoms *(r = 0.13, p = .012)*, and negatively correlated with resistance to peer influence *(r = -0.13, p = .013)*. Local perceived social status significantly moderated the relationship between PSRS scores and depressive symptoms *($\beta$ = 0.005, SE = 0.002, t = 2.36, p = .019)*. A general sense of belonging did not moderate this relationship, but feelings of rejection and exclusion were directly linked to higher perceived social risks *($\beta$ = -0.112, SE = 0.045, t = -2.48, p = .014)*."**

**Introduction**

15. Could the authors please add more detailed description of the anticipated applications of

such a tool? While broad suggestions of clinical and research potential are made, it is still unclear how the new tool may be used, particularly clinically.

We thank the reviewer for the comment, we have now added in more detail to where we believe that the PSRS could be used, and comment on its utility in clinical practise:

**“Future research should explore these distinct elements of social risk in more diverse and representative samples to enhance the generalisability of the findings. If future research establishes robust psychometric properties of the PSRS in clinical contexts, clinicians may eventually consider using it to better understand social risk perceptions that contribute to maladaptive behaviours or distress. For example, clinicians could use the PSRS to identify individuals with heightened sensitivity to social risks, a feature often linked to conditions such as social anxiety, depression, and interpersonal difficulties. This information could guide therapeutic approaches by highlighting specific risk perceptions that contribute to maladaptive behaviours or distress. However, until then, the PSRS should be regarded primarily as a research measure rather than a clinical assessment instrument.”**

16. Could the authors please describe how they ensured that “the items included in our scale reflect everyday social scenarios that people from diverse backgrounds can understand and evaluate”? This step of the research isn’t covered in the methods or results.

We have now included in more detail how we created the items in line with past measurements, and how we aimed to ensure for everyday usage:

**”Items were constructed to reflect a wide array of social risk behaviours. For instance, social risk items included statements such as: "Telling a risky joke” (approach-related social risk), "Wearing clothes that your friends wouldn't approve of“ (norm-violating social risk), “Not contributing to a group gift that you find too expensive” (avoidance-related social risk), and “Refusing to spread a rumour, even when all your friends are doing it” (norm-violating social risk). These items were designed to tap into different constructs of social risk, ensuring a comprehensive measure. Finally, items were carefully crafted using clear**

**and accessible language, avoiding jargon or culturally specific references. This approach prioritised scenarios that were broadly relatable, such as "Not laughing at a group's inside joke that you don't find funny," rather than situations tied to specific cultural norms. As such, a list of 36 items were developed and created to create a list of social risk scenarios that could tap into distinct constructs of social risk-taking**."

**Limitations:**

**"One limitation of our measure is that, although we endeavoured to construct items that were broadly relevant and accessible by avoiding jargon or culturally specific references, these steps were not systematically tested for applicability across different cultures or demographic groups. While the scenarios were designed to reflect everyday social situations, it is possible that certain items may still be influenced by cultural norms, language nuances, or social contexts that were not fully captured in our sample. Consequently, future research should undertake cross-cultural validation and further diversify sampling to confirm the scale's relevance and comprehensiveness in more varied populations. "**

**Methods**

17. In several places in the manuscript it is stated that people from diverse backgrounds were recruited, yet no sampling strategy is described and no information confirming the diversity (or even defining what diversity is meant here). The lack of a sampling strategy is a major limitation, raises questions of regarding bias, and means that disaggregation of results by characteristics such as sex is probably not possible. The lack of a description of the study sample means it is very unclear who was in the sample and who the developed tool applies to.

In the revised manuscript, we have clarified our sampling strategy and provided a detailed description of the study sample. We have also included more detail into our limitation section, where we acknowledge that caution should be applied outside of the demographic that we used. We have also included greater detail in terms of Ethnicity and the Socioeconomic stauts of our participants in the CFA.

**Added to the sample size section:**

**"Sample size for our EFA was determined based on Kaiser-Meyer-Olkin index and the Bartlett's test, with scores above .07 and below .05, indicating a suitable sample size, respectively. Our CFA sample size was calculated *a priori* and pre-registered. We aimed for a minimum of 360 participants (10 participants per question included in the EFA). In practice we overrecruited to account for attrition and poor responding[35 36 37]."**

**Added to Sample 2:**

**"The majority identified as White (80.2%), followed by Asian, including Pacific Islander (8.1%), Biracial or Multiracial (5.3%), Black or African American (5.1%), and Hispanic (1.3%). Regarding socioeconomic status, 13.5% of participants reported an annual household income of less than £11,999, 25.4% reported £12,000–£24,999, 44.7% fell within £25,000–£49,999, 11.4% reported incomes of £50,000–£74,999, and 2.3% had an income of £75,000 or more. Additionally, 2.7% of participants preferred not to disclose their income."**

**Added to the discussion:**

**"**Although the factor structure identified through exploratory and confirmatory factor analyses appears robust, it is crucial for future research to replicate these findings in different and more diverse samples. **Replication studies will help confirm the generalisability and stability of the factor structure across various populations."**

18. Could the authors describe how the DASS short version was chosen out of the many short form screening tools for Depression?

We have now added in more detail to discuss why we chose the DASS-7:

**"The DASS-7 was selected for its reliability in measuring depressive symptoms and its ability to differentiate depressed mood from anxiety and stress. The conciseness and**

**empirical validity of the DASS-7 ensured for a reduction in participant burden, making it well-suited for online studies. Additionally, its use in prior research on rejection sensitivity and depression[31] ensures methodological consistency and comparability, in which we also administered the Rejection Sensitivity Questionnaire ($O^2S^3$) from the same source to maintain comparability of measures."**

19. Could the authors please describe the rationale for conducting the EFA in a sample of 18-25 year olds and then the CFA in a wider age group (18-65 year olds)? Are there any potential issues with this difference in samples?

We thank the reviewer for raising this point. We have discussed further in the manuscript our rationale for this. To address this, we have ICC tested based on age across our EFA and CFA sample. The results showed excellent consistency across samples by age. Please refer to our response to point 7 above.

**Results**

20. Could the authors give an age breakdown for the sample involved in CFA?

We have now included an age breakdown in the sample involved in the CFA:

***Sample 2*: Confirmatory Factor Analysis (CFA) and validation**

**Participants (N = 389) were recruited from Prolific Academic, all residing in the UK. Participants (243 Women, 143 Men, 3 non-binary) were aged 18–65 years (Mean age = 39.33, SD = 11.68).**

21. Could the authors also give the age and sex breakdown for the sample who took part in the test-retest reliability phase?

We have now included an age and sex breakdown in the sample involved in the test-retest:

**"To measure the test-retest reliability of the PSRS, 108 adult participants were invited to complete the questionnaire a second time 13-15 days later; 108 participants responded.**

**Mean age = 40.72, SD age = 11.17; 42 were Male, 66 were Female. Test–retest reliability for the overall scale across the interval was good, *ICC = 0.70, (95% CI [0.571, 0.797])*. This confirms the stability of the scale items over time.”**

**Discussion and Conclusion**

22. It is concluded that the “Perceived Social Risk Scale (PSRS) offers a reliable and valid measure to assess perceptions of social risk-taking” but without a representative sample of the population, and without a description of the study sample, we don’t know who this measure is reliable or valid for.

We thank the reviewer for this comment. We have now added in some nuance to this, and explained that future research will be needed to highlight its clinical use. E.g.,:

**“The PSRS provides a valuable tool for researchers to assess an individual’s perception of social risk-taking. By distilling social risk into discrete sub-categories, our scale facilitates the measurement of the specific social risks individuals perceive and the factors that influence these perceptions.”**

**“Replication studies will help confirm the generalisability and stability of the factor structure across various populations. In addition, the PSRS was not validated in a younger adolescent population. Given that adolescence is a sensitive period for social development and risk-taking behaviours, future studies should focus on validating the PSRS in adolescent samples. This would help determine the scale's applicability and relevance for younger individuals.”**
